# Isosorbide Fatty Acid Diesters Have Synergistic Anti-Inflammatory Effects in Cytokine-Induced Tissue Culture Models of Atopic Dermatitis

**DOI:** 10.3390/ijms232214307

**Published:** 2022-11-18

**Authors:** William R. Swindell, Krzysztof Bojanowski, Ratan K. Chaudhuri

**Affiliations:** 1 Department of Internal Medicine, University of Texas Southwestern Medical Center, Dallas, TX 75390, USA; 2 Sunny BioDiscovery Inc., Santa Paula, CA 93060, USA; 3 Sytheon Ltd., Parsippany, NJ 07054, USA

**Keywords:** atopic dermatitis, drug development, emollient, isosorbide diesters, hypersensitivity, moisturizer, skin substitute, topical therapy

## Abstract

Atopic dermatitis (AD) is a chronic disease in which epidermal barrier disruption triggers Th2-mediated eruption of eczematous lesions. Topical emollients are a cornerstone of chronic management. This study evaluated efficacy of two plant-derived oil derivatives, isosorbide di-(linoleate/oleate) (IDL) and isosorbide dicaprylate (IDC), using AD-like tissue culture models. Treatment of reconstituted human epidermis with cytokine cocktail (IL-4 + IL-13 + TNF-α + IL-31) compromised the epidermal barrier, but this was prevented by co-treatment with IDL and IDC. Cytokine stimulation also dysregulated expression of keratinocyte (KC) differentiation genes whereas treatment with IDC or IDL + IDC up-regulated genes associated with early (but not late) KC differentiation. Although neither IDL nor IDC inhibited Th2 cytokine responses, both compounds repressed TNF-α-induced genes and IDL + IDC led to synergistic down-regulation of inflammatory (*IL1B*, *ITGA5*) and neurogenic pruritus (*TRPA1*) mediators. Treatment of cytokine-stimulated skin explants with IDC decreased lactate dehydrogenase (LDH) secretion by more than 50% (more than observed with cyclosporine) and in vitro LDH activity was inhibited by IDL and IDC. These results demonstrate anti-inflammatory mechanisms of isosorbide fatty acid diesters in AD-like skin models. Our findings highlight the multifunctional potential of plant oil derivatives as topical ingredients and support studies of IDL and IDC as therapeutic candidates.

## 1. Introduction

Atopic dermatitis (AD) is a prevalent skin disease characterized by immune dysregulation and barrier function abnormalities resulting in cutaneous water loss [1,2]. This leads to pruritic skin eruptions that commonly occur on skin flexures, typically starting in early childhood, but then continuing throughout adult life [1,2]. AD has a complex genetic basis and develops from an interaction between genetic and environmental factors, with disruption of the epidermal barrier viewed as a triggering event that initiates a Th2-dominant inflammatory cascade [3]. Disease flares are treated with topical steroids, calcineurin inhibitors or phosphodiesterase-4 inhibitors (i.e., crisaborole), but the cornerstone of long-term therapy includes regular application of emollients to promote barrier repair and retention of skin hydration [4]. Topical agents such as petrolatum or lanolin serve an occlusive function, preventing water loss by providing a physical barrier to facilitate endogenous healing [5]. These agents are often combined with humectants such as glycerin, lactic acid, and panthenol to bind water molecules within epidermal layers [6]. Alternatively, lipid-based compositions feature a physiological balance of ceramides, cholesterol and free fatty acids, which are directly delivered to the *stratum corneum* with topical application [7]. Next-generation topical creams have also been formulated to include an all-in-one mix of ingredients, together designed to restore the skin barrier but also repress inflammation, itching and bacterial growth [8]. Increasingly, therefore, a spectrum of topical products is available for chronic AD management, which may be used alongside systemic immunosuppressive therapy for patients with severe disease [1,2].

Plant-derived oils can improve barrier function through delivery of essential fatty acids but are multifunctional as well, with anti-inflammatory and anti-bacterial effects [9,10,11]. Along these lines, isosorbide di-(linoleate/oleate) (IDL) and isosorbide dicaprylate (IDC) are recently developed isosorbide diesters with clinical efficacy for improving skin hydration and inhibiting transepidermal water loss (TEWL) [12,13]. Isosorbide di-(linoleate/oleate) (IDL) is an isosorbide diester generated by esterifying isosorbide with sunflower fatty acids [12]. In cultured keratinocytes, IDL had pro-differentiation effects and increased abundance of barrier proteins such as filaggrin (FLG) and involucrin (IVL) [12]. Consistent with this, IDL improved skin hydration and decreased TEWL in human subjects with dry skin [12]. IDC is an ester of isosorbide and octanoic (caprylic) acid with demonstrated advantages over alternative agents such as glycerol [13]. IDC improved skin hydration more than glycerol, and the combination of IDC with glycerol improved skin hydration more than glycerol alone [13]. These effects were associated with up-regulated expression of aquaporin 3 (*AQP3*), CD44 molecule (*CD44*), E-cadherin (*CDH1*) and genes involved in keratinocyte (KC) differentiation (e.g., *LCE1E*, *LCE3D*, *CERS3*, *SPRR3*) [13]. Both IDL and IDC improve skin hydration through barrier repair, but a distinguishing feature of IDL may be its anti-inflammatory activity, which involves down-regulation of T cell activated genes and protection of *stratum corneum* against cytokine-induced degradation [12]. Similar anti-inflammatory effects were not previously described as part of the IDC activity spectrum [13].

Two- or three-dimensional in vitro models provide tools for AD drug development as systems for rapid screening to quickly evaluate potential AD topical therapies [14]. Recent work has developed cytokine cocktails to activate epidermal and/or inflammatory cells, thereby replicating some histological and immunological features of AD, such as spongiosis, apoptosis, altered lipid organization, and augmented production of thymic stromal lymphopoietin (TSLP) [15,16]. Th2 cytokines such as IL-4 and IL-13 are central components of such cocktails and sufficient to induce spongiosis, apoptosis and other disease-specific features of AD skin [17]. These cytokines alone, however, do not fully replicate the loss of differentiation, inflammatory cascades and pruritus-associated reactions that occur in AD skin [18]. IL-4 and IL-13 have thus been combined with other immunostimulating agents, which appear to yield AD-like changes in KC differentiation (IL-25) [19], inflammation (TNF-α, IL-1α) [20], pruritus reaction (IL-31) [16], KC activation (IL-22) [21] and innate immune response (Poly I:C) [22]. Although the optimal cytokine cocktail has not been established, insights into AD-like therapeutic responses have been obtained by combining IL-4 and IL-13 with one or more of the above-mentioned stimulating agents [23,24].

This study used cytokine-stimulated skin culture models to evaluate effects of two isosorbide fatty acid diesters (IDL and IDC) at the histological and molecular levels. Our experiments utilize reconstructed human epidermis (RHE) and ex vivo skin biopsy cultures treated with Th2 cytokines (IL-4 + IL-13 ± IL-5) and TNF-α. These combinations of Th2 and pro-inflammatory cytokines were previously shown to induce features of AD skin, including spongiosis, TSLP production, and alterations in KC differentiation and *stratum corneum* lipid composition [15,16]. Our results provide further evaluation of the Th2 cytokine + TNF-α approach as a model system for studying AD-like responses in three-dimensional skin cultures. Our findings also evaluate mechanisms by which isosorbide fatty acid diesters may influence barrier integrity and inflammatory response to barrier compromise.

## 2. Results

### 2.1. IDL and IDC Prevent Cytokine-Induced Disruption of Epidermal Morphology

**Figure 1:**
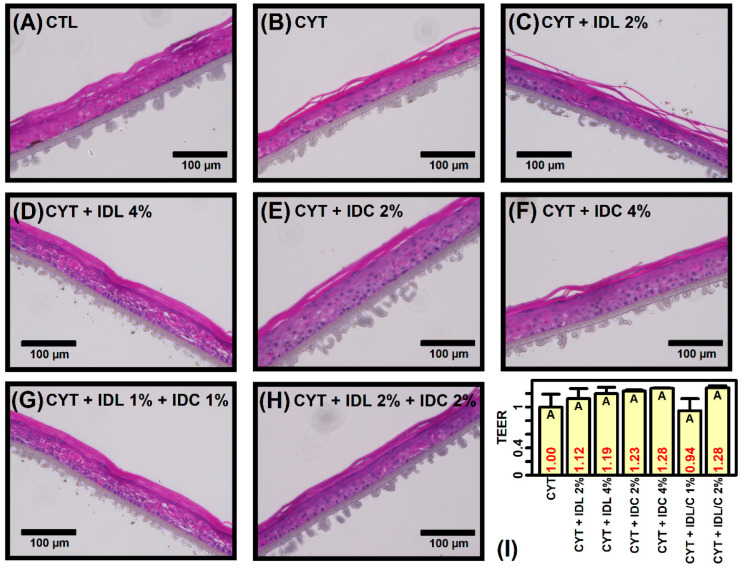
H&E stains and transepithelial electrical resistance (TEER) measurements. (**A**–**H**) H&E stains. Tissues were treated with vehicle only (CTL) or cytokine cocktail (IL-4, IL-13, IL-31 and TNF-α) with or without test compounds (IDL or IDC) at different concentrations. (**I**) TEER measurements. The average value of 3 replicates per group is shown (±1 standard error). Groups without the same letter differ significantly (*p* < 0.05, Fisher’s least significant difference).

Premature reconstituted human epidermis (RHE) tissues were treated with cytokine cocktail (IL-4, IL-13, TNF-α and IL-31) for 1 week and tissue structure was evaluated by H&E staining (Figure 1). Disruption of epidermal differentiation with a fissured tissue architecture was apparent (Figure 1B). High-dose IDL (4%) improved tissue architecture more so than low-dose IDL (2%) (Figure 1C,D). Treatment with IDC alone increased tissue integrity as well but only partially (Figure 1E,F). Tissue structure appeared largely preserved when IDL and IDC were applied in combination (Figure 1G,H). Compared to tissue treated with cytokine only, the addition of IDL or IDC increased transepithelial electrical resistance (TEER) by 12–28% although this effect was non-significant (*p* > 0.05, Figure 1I). High-dose (2%) combination IDL + IDC increased TEER by 28% whereas TEER did not increase following low-dose (1%) combination (Figure 1I).

### 2.2. Cytokines Induce Inflammatory and Mitochondrial Gene Expression but Repress Epidermal Development and KC Differentiation Pathways

**Figure 2:**
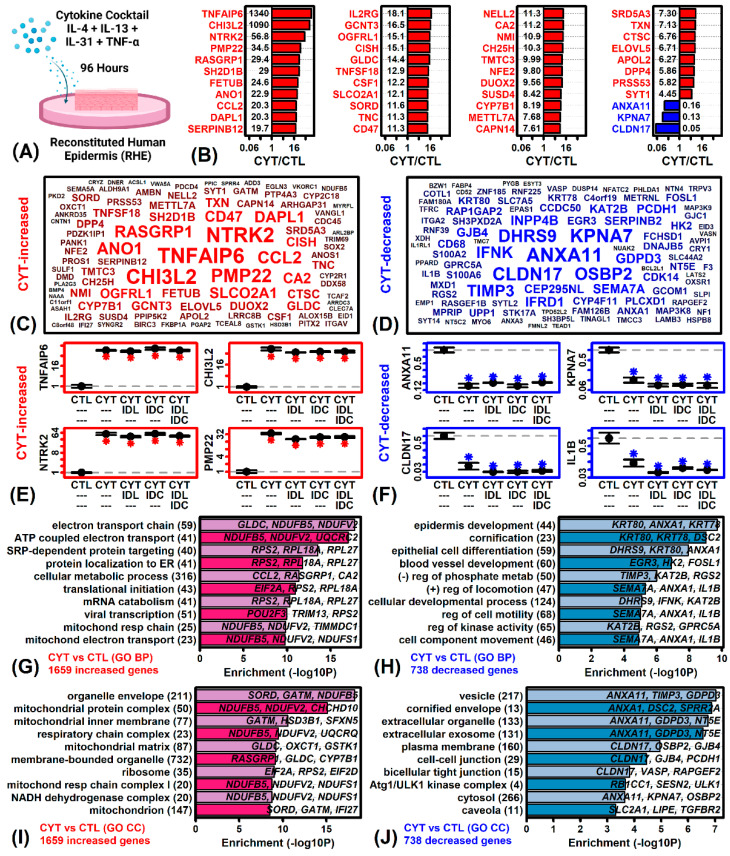
Gene expression response to cytokine cocktail (IL-4, IL-13, IL-31 and TNF-α) in RHE tissue. (**A**) Experimental design. (**B**) Top 44 DEGs with lowest *p*-value (ranked according to fold-change). (**C**) Top 100 cytokine-increased DEGs. (**D**) Top 100 cytokine-decreased DEGs. In (**C**,**D**), genes with larger fonts and blue/red color have lower *p*-values. (**E**) Cytokine-increased DEG average expression. (**F**) Cytokine-decreased DEG average expression. In (**E**,**F**), expression scores are normalized to the CTL treatment and an asterisk (*) is used to indicate significant differences compared to the CTL treatment (*p* < 0.05, two-sample *t*-test). (**G**,**I**) GO BP and CC terms enriched among cytokine-increased DEGs. (**H**,**J**) GO BP and CC terms enriched among cytokine-decreased DEGs. In (**G**–**J**), the number of DEGs associated with each GO term is indicated in parentheses and example genes are listed within each figure.

Microarrays were used to evaluate gene expression in RHE tissues exposed to cytokine cocktail over a 96 h period (Figure 2A). As expected, cytokine treatment led to large changes in gene expression, with 2397 genes significantly altered (1659 CYT-increased, 738 CYT-decreased; FDR < 0.10, FC > 1.50 or FC < 0.67). Genes most strongly increased by this cocktail included TNF alpha induced protein 6 (*TNFAIP6*), chitinase 3 like 2 (*CHI3L2*), neurotrophic receptor tyrosine kinase 2 (*NTRK2*) and peripheral myelin protein 22 (*PMP22*) (Figure 2B,C,E). Cytokine-increased genes were associated with electron transport, protein localization and the mitochondrial membrane (Figure 2G,I). Such genes were also associated with type I interferon response and the intrinsic apoptotic signaling pathway. Genes most strongly decreased by the cocktail included annexin A11 (*ANXA11*), karyopherin alpha 7 (*KPNA7*), claudin 17 (*CLDN17*) and interleukin 1 beta (*IL1B*) (Figure 2B,D,F). Cytokine-decreased genes were associated with epidermis development, cornification and vesicles (Figure 2H,J). Such genes were also associated with lipid homeostasis and keratinocyte differentiation.

### 2.3. The Cytokine Cocktail Induces an AD-like Gene Expression Response in RHE Tissue Cultures

Cytokine-regulated genes were compared to the meta-analysis derived atopic dermatitis (MADAD) transcriptome, which comprises a set of genes robustly increased or decreased in atopic dermatitis lesions compared to non-lesional skin [25]. We identified 293 MADAD-increased and 129 MADAD-decreased genes for which expression was sufficiently detectable to be included in the CYT vs. CTL differential expression analysis. Of the 293 MADAD-increased genes, 69 were elevated by cytokine treatment in RHE skin (e.g., *CHI3L2*, *RASGRP1*, *CCL2*; *p* = 1.8 × 10^−0^; Figure S1A,C). Likewise, of the 129 MADAD-decreased genes, 13 were repressed by cytokine treatment in RHE skin (e.g., *GPRC5A*, *TIMP3*, *CLIC5*; *p* = 0.0065; Figures S1B,D). On average, the 293 MADAD-increased genes were increased by 22%, corresponding to an average FC (CYT/CTL) significantly greater than that seen in randomly sampled gene sets (*p* < 0.001; Figure S1E). The 129 MADAD-decreased genes were decreased by 15% on average, corresponding to an average FC significantly lower than that seen in randomly sampled gene sets (*p* < 0.001; Figure S1F). Consistent with these findings, a significant majority of MADAD-increased genes were CYT-increased (*p* < 0.001, Figure S1G), whereas a significant majority of MADAD-decreased genes were CYT-decreased (*p* < 0.001, Figure S1H). Additionally, when all RHE-expressed genes were ranked according to their cytokine response, MADAD-increased genes were enriched in the top part of the list (i.e., among CYT-increased genes; *p* = 0.001; Figure S1I), while MADAD-decreased genes were significantly enriched near the bottom of the list (i.e., among CYT-decreased genes; *p* = 5.41 × 10^−0.9^; Figure S1J).

### 2.4. IDL, IDC and IDL + IDC Up-Regulate Cell Cycle Genes and Decrease Expression of Genes Associated with Development and Differentiation

Microarrays were next used to evaluate effects of test compounds (IDL, IDC and IDL + IDC in cytokine-treated RHE tissues. Smaller changes in gene expression were seen, as compared to the magnitude of cytokine response (when compared to untreated tissue), and thus a less stringent significance threshold was adopted (*p* < 0.05, FC > 1.50 or FC < 0.67). Given these criteria, IDL and IDC altered the expression of 611 and 1125 DEGs, respectively, whereas the IDL + IDC combination altered expression of 684 DEGs.

**Figure 3:**
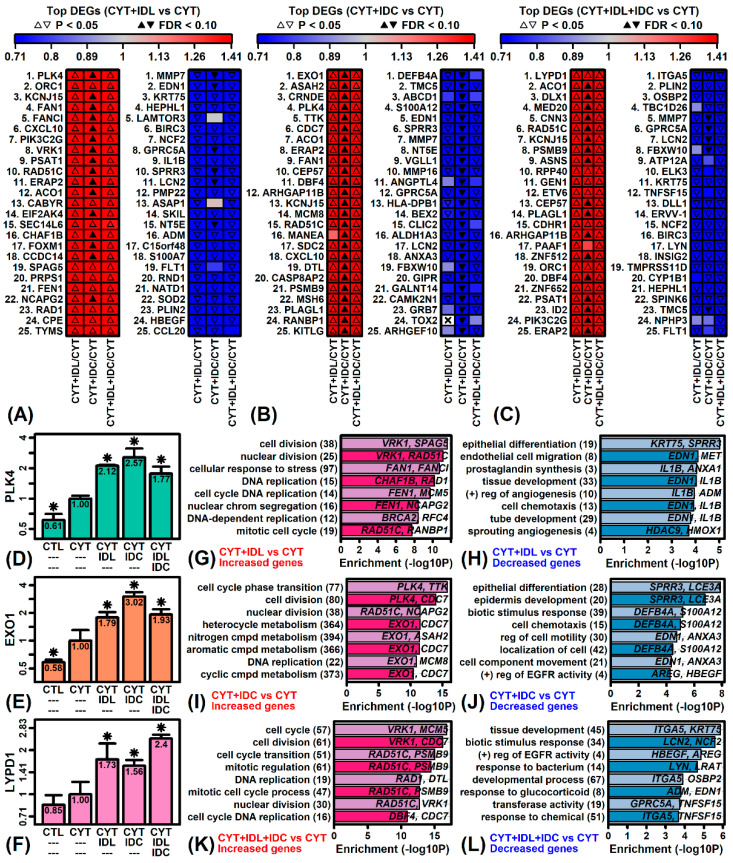
Gene expression responses to IDL, IDC and IDL + IDC in cytokine-stimulated RHE tissue. (**A**–**C**) Top-ranked DEGs. Heatmaps show the top 25 increased and decreased DEGs for each comparison. (**D**) *PLK4*. (**E**) *EXO1*. (**F**) *LYDP1*. In (**D**–**F**), average expression is shown for each treatment (* *p* < 0.05, moderated *t*-test, comparison to CYT). (**G**–**L**) GO BP terms. Figures show Gene Ontology (GO) biological process (BP) terms most strongly enriched among DEGs with altered expression in each comparison (*p* < 0.05, FC > 1.50 or FC < 0.67). The degree of enrichment is shown on the horizontal axis (i.e., −log10-transformed *p*-value). The number of DEGs associated with each GO BP term is given in parentheses. Example DEGs associated with each term are listed within figures.

Genes increased most strongly by IDL included polo like kinase 4 (*PLK4*), origin recognition complex subunit 1 (*ORC1*), and potassium inwardly rectifying channel subfamily J member 15 (*KCNJ15*) (Figure 3A,D), and such genes were most strongly associated cell division, nuclear division and stress response (Figure 3G). Genes most strongly decreased by IDL included matrix metallopeptidase 7 (*MMP7*), endothelin 1 (*EDN1*), and keratin 75 (*KRT75*) (Figure 3A) and such genes were most strongly associated with epithelial differentiation, cell migration and prostaglandin synthesis (Figure 3H).

Genes most strongly increased by IDC included exonuclease 1 (*EXO1*), N-acylsphingosine amidohydrolase 2 (*ASAH2*), and colorectal neoplasia differentially expressed (*CRNDE*) (Figure 3B,E) and such genes were most strongly associated with cell cycle phase transition, cell division and nuclear division (Figure 3I). Genes most strongly decreased by IDC included defensin beta 4A (*DEFB4A*), transmembrane channel like 5 (*TMC5*), and ATP binding cassette subfamily D member 1 (*ABCD1*) (Figure 3B) and such genes were most strongly associated with epithelial differentiation, epidermis development, and response to biotic stimulus (Figure 3J).

Genes most strongly increased by IDL + IDC included LY6/PLAUR domain containing 1 (*LYPD1*), aconitase 1 (*ACO1*), and distal-less homeobox 1 (*DLX1*) (Figure 3C,F) and such genes were most strongly associated with cell cycle, cell division, and cell cycle transition (Figure 3K). Genes most strongly decreased by IDL + IDC included integrin subunit alpha 5 (*ITGA5*), perilipin 2 (*PLIN2*), and oxysterol binding protein 2 (*OSBP2*) and were associated with tissue development, response to biotic stimulus, and positive regulation of EGFR activity (Figure 3L).

### 2.5. IDL, IDC and IDL + IDC Increase Expression of Basal Layer and Early KC Differentiation Genes but Repress Expression of Genes Associated with Late KC Differentiation

Genes down-regulated by the cytokine cocktail and by IDL, IDC and IDL + IDC were each associated with differentiation or development (Figure 2H,J and Figure 3H,J,L). Consistent with this, late differentiation genes such as *IVL* and *TGM1* were significantly decreased by the cytokine cocktail (FDR < 0.10; Figure S2A), and such genes likewise trended towards decreased expression after treatment with IDL, IDC and IDL + IDC (Figure S2E,I,M). On the other hand, these treatments increased expression of marker genes associated with the basal layer and early differentiation (Figure S2E,I,M).

We next evaluated the expression of genes induced by differentiation over 7 days during a regenerated epidermis time course (GSE52651) [26]. There was no clear trend towards increased or decreased expression of such genes by the cytokine cocktail (Figure S2B–D). However, such genes tended to be down-regulated by IDL (Figure S2F–H), IDC (Figure S2J–L) and IDL + IDC (Figure S2N–P).

### 2.6. IDL, IDC and IDL + IDC Increase Expression of Genes Associated with Late but Not Early Interphase

Genes up-regulated by IDL, IDC and IDL + IDC were associated with the cell cycle (Figure 3G,I,K). We thus evaluated the expression of genes associated with different cell cycle phases [27]. The cytokine cocktail tended to increase expression of genes associated with each cell cycle phase although the strongest increase was observed among genes associated with late interphase (G2) (*p* < 0.05; Figure S3A). Likewise, IDL, IDC and IDL + IDC each most strongly up-regulated expression of late-interphase (G2) genes (*p* < 0.05; Figure S3B–D). In contrast, G1 phase genes were not systematically increased by cytokines or most treatments (except IDC; Figure S3E), whereas S, G2 and M phase genes were all biased towards increased expression by cytokines and each treatment (*p* < 0.05; Figure S3F–H).

### 2.7. IDL, IDC and IDL + IDC Oppose Gene Expression Responses Associated with TNF-α but Not Th2 Cytokines (IL-4, IL-13, IL-31)

**Figure 4:**
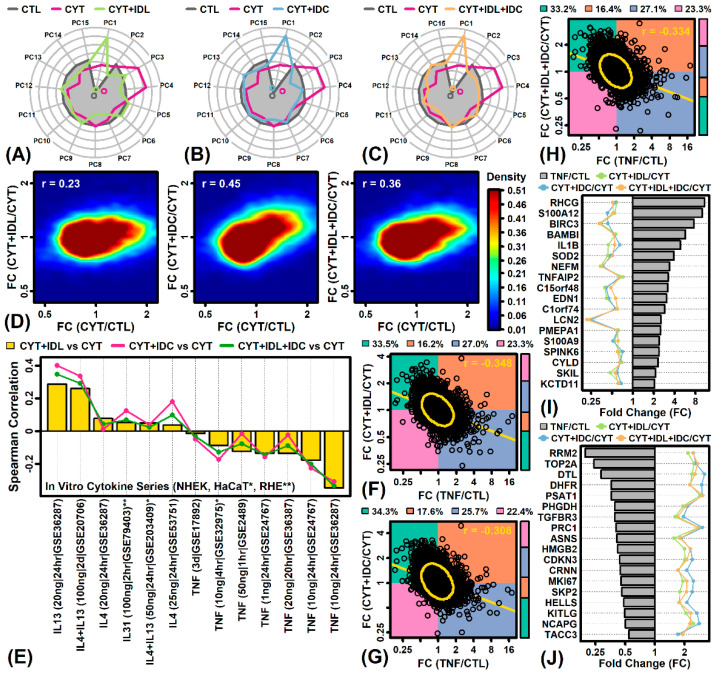
Comparison to Th2 and TNF-α cytokine responses. (**A**–**C**) PC radial plots. Average PC scores are plotted for CTL and CYT samples along with those for each experimental treatment (CYT + IDL, CYT + IDC, CYT + IDL + IDC). (**D**) Gene density scatterplots. Scatterplots compare FC estimates between treatment and cytokine responses. (**E**) In vitro Th2 and TNF-α cytokine series. Fold-change estimates were compared to those observed in experiments in which normal human epidermal keratinocytes (NHEKs), HaCaT (*), or RHE (**) were treated with cytokines. The spearman correlation between FC estimates is shown for each comparison. The cytokine dose, treatment duration, and GEO accession number is given (**bottom margin**). (**F**–**H**) Scatterplot comparisons to TNF responses (GSE36287). Each point represents an individual gene. The proportion of genes within each quadrant is indicated (**top margin**) and represented by the sidebar. The spearman rank correlation is shown (**top right**). The yellow ellipse outlines the middle 90% of genes closest to the bivariate centroid (Mahalanobis distance). The least-squares regression line is shown (yellow). (**I**) TNF-increased genes down-regulated by experimental treatments. (**J**) TNF-decreased genes up-regulated by experimental treatments. In (**I**,**J**), each gene was increased or decreased significantly by treatment of primary human KCs with TNF (10 ng/mL) for 24 h (FDR < 0.10, GSE36287). FC estimates are shown for each experimental treatment (see **top margin legend**).

Treatment of cytokine-stimulated RHE with IDL, IDC or IDL + IDC shifted expression patterns compared to that seen in RHE treated with cytokines alone. Treatment of RHE with IDL, IDC or IDL + IDC increased scores with respect to the first principal component axis, but decreased scores with respect to the 3rd and 4th axes (Figure 4A–C). Broadly, the effects of IDL, IDC and IDL + IDC were correlated with cytokine-induced expression shifts (Figure 4D). To dissect out a finer pattern, however, responses to IDL, IDC and IDL + IDC were compared to those in KCs or RHE tissue treated with Th2 cytokines (IL-4, IL-13, IL-31) and TNF-α (Figure 4E). This showed a clear trend in which effects of IDL, IDC and IDL + IDC were either non-correlated or positively correlated with Th2 cytokine responses, but were non-correlated or negatively correlated with TNF-α responses (Figure 4E). With regard to one TNF-α experiment (GSE36287) [28], for example, the genome-wide correlation between IDL, IDC and IDL + IDC responses with that of TNF-α was less than −0.30 in each case (*p* < 0.05; Figure 4F–H). Genes up-regulated by TNF-α in KCs (FDR < 0.10) but down-regulated by IDL, IDC and IDL + IDC included Rh family C glycoprotein (*RHCG*), S100 calcium binding protein A12 (*S100A12*), and baculoviral IAP repeat containing 3 (*BIRC3*) (Figure 4I). Genes down-regulated by TNF-α in KCs (FDR < 0.10) but up-regulated by IDL, IDC and IDL + IDC included ribonucleotide reductase M2 polypeptide (*RRM2*), DNA topoisomerase II alpha (*TOP2A*), and denticleless E3 ubiquitin protein ligase homolog (*DTL*) (Figure 4J).

### 2.8. IDL and IDC Synergistically Repress Expression of Pro-Inflammatory Mediators (IL1B, ITGA5, TRPA1)

**Figure 5:**
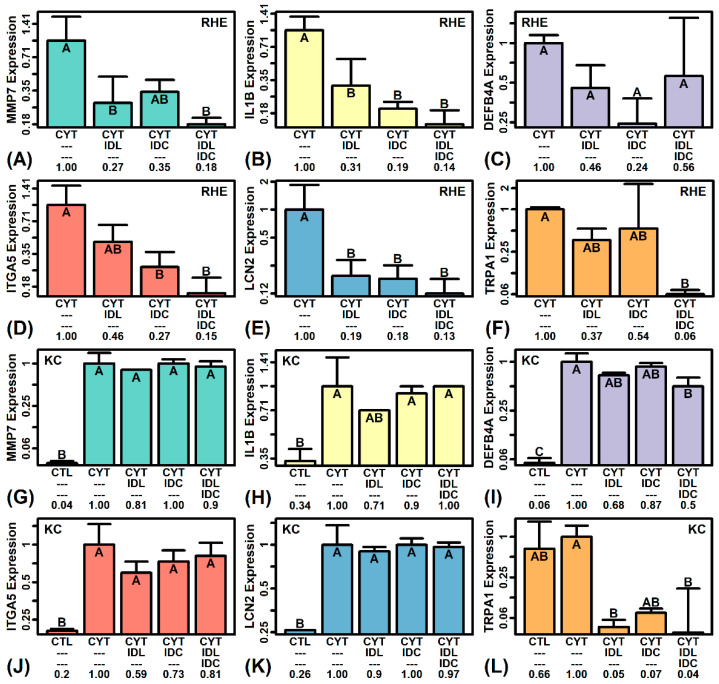
RT-PCR analyses. (**A**,**G**) *MMP7*. (**B**,**H**) *IL1B*. (**C**,**I**) *DEFB4A*. (**D**,**J**) *ITGA5*. (**E**,**K**) *LCN2*. (**F**,**L**) *TRPA1*. Experiments were performed using RHE tissue (**A**–**F**, *n* = 3 per treatment) and HaCaT KCs (**G**–**L**, *n* = 2 per treatment). Average relative expression of each gene is shown (± 1 standard error). Groups without the same letter differ significantly (*p* < 0.05, Fisher’s least significant difference (LSD)). The average FC for each group is listed (**bottom margin**). Expression of 18S ribosomal RNA (18S) was used a reference. For RHE experiments (**A**–**F**), analyses were performed using the same RNA samples analyzed by microarray.

RT-PCR was used to further evaluate the expression of selected genes down-regulated by IDL and/or IDC in microarray analyses of cytokine-treated RHE (i.e., *MMP7*, *IL1B*, *DEFB4A*, *ITGA5*, *LCN2*; see Figure 3). In most cases, the trend towards decreased expression was confirmed using RT-PCR (*p* < 0.05), with lowest expression seen in RHE treated with the IDL + IDC combination (Figure 5A,B,D–F). The one exception was *DEFB4A*, for which we did not observe significant down-regulation by RT-PCR in RHE tissue (*p* > 0.05, Figure 5C). However, when the experiment was repeated using HaCaT KCs, *DEFB4A* expression (evaluated by RT-PCR) was significantly decreased by IDL + IDC (*p* < 0.05, Figure 5I). Otherwise, neither IDL or IDC significantly altered expression of genes examined in HaCaT cells (*p* > 0.05; Figure 5G,H,J–L).

Transient receptor potential cation channel subfamily A member 1 (*TRPA1*) was not included in differential expression analyses due to its low expression (below the limits of detection by microarray). We thus evaluated *TRPA1* expression by RT-PCR, which showed that its expression was decreased by both IDL and IDC, with significant down-regulation (>90%) by IDL + IDC (*p* < 0.05, Figure 5F). Consistent with this, IDL, IDC and IDL + IDC all decreased *TRPA1* expression more than 90% in HaCaT KCs, although significant down-regulation was only observed with IDL + IDC treatment (*p* < 0.05, Figure 5L).

### 2.9. The IDL + IDC Anti-Inflammatory Response More Closely Resembles That of a Calcineurin Inhibitor Rather Than Corticosteroid

Topical anti-inflammatory treatments such as corticosteroids or calcineurin inhibitor are first-line treatments for AD management [29,30,31]. Genes altered by IDL + IDC were thus compared to those altered by 3 weeks of treatment with betamethasone (BET) or pimecrolimus (PIM) in lesional skin from AD patients [32]. We could identify genes increased by both IDL + IDC and BET (e.g., *CHEK1*, *DEGS1*, *ACAT1*; *p* < 0.05; Figure S4A), as well as genes decreased by both treatments (e.g., *BIRC3*, *TGM3*, *SOD2*; *p* < 0.05; Figure S4B). Overall, however, there was no significant overlap or association between IDL + IDC and BET expression responses (*p* > 0.05; Figure S4C–J). We identified several genes increased by both IDL + IDC and PIM (e.g., *CDHR1*, *SRGAP2*, *NPR3*; *p* < 0.05; Figure S5A) but there was no significant overlap between genes increased by both treatments (*p* > 0.05; Figure S5C,E,G,I). However, there was significant overlap between genes decreased by IDL + IDC and PIM (*p* = 0.005, Figure S5D), although the association was only marginally significant in some analyses (*p* ≤ 0.16; Figure S5F,H. Genes decreased by both IDL + IDC and PIM included *IL36G*, *S100A9* and *CCL1* (*p* < 0.05; Figure S5B). Such genes frequently localized to the membrane and were associated with innate immune response and response to cytokine (Figure S5K,L).

### 2.10. IDL, IDC and IDL + IDC Alter the Expression of Pruritus-Associated Genes

Pruritus is an important feature of AD that contributes to barrier compromise and disease exacerbations [33]. We therefore evaluated effects of IDL, IDC and IDL + IDC on a set of 130 pruritus-associated genes identified from the Human Phenotype Ontology database (ontology term identifier HP:0000989) [34]. We identified several pruritus-associated genes increased by IDL, IDC and IDL + IDC (e.g., *BRCA2*, *KRT5*, *TCF4*; Figure S6A), as well as several such genes decreased by IDL, IDC and/or IDL + IDC (e.g., *IL2RG*, *TRPV3*, *TNFSF15*, *IL17RC*; Figure S6A,F–I). As a group, pruritus-associated genes were not significantly more likely to be increased or decreased by cytokine stimulation, IDL or IDC (*p* ≥ 0.078; Figure S6B–D). However, such genes were significantly more likely to be down-regulated by IDL + IDC as compared to other randomly sampled gene sets of the same size (*p* = 0.024; Figure S6E).

### 2.11. IDC Decreases LDH Secretion by more Than 50% in Cytokine-Treated Skin Explants

**Figure 6:**
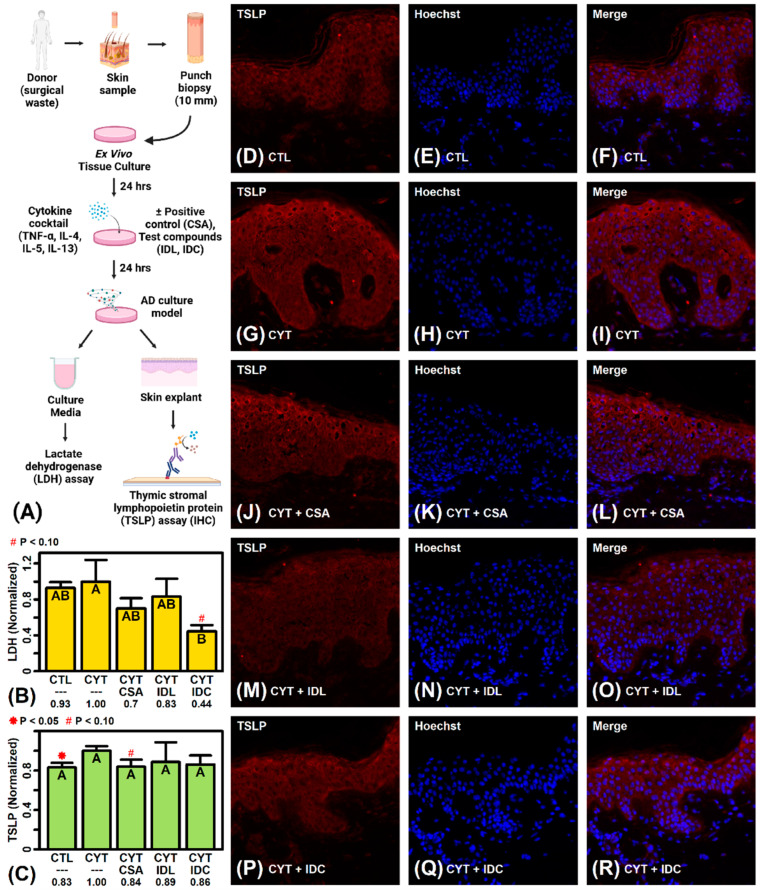
Effect of IDL and IDC on cell survival and inflammation in an AD explant model. (**A**) Experimental design. (**B**) Lactate dehydrogenase (LDH). (**C**) Thymic stromal lymphopoietin protein (TSLP). In (**B**,**C**), the average value is shown for each group (*n* = 3 per treatment). Treatments that do not share the same letter differ significantly (*p* < 0.05, Fisher’s least significant difference). Results from one-tailed two-sample *t*-tests are also shown (top margin symbols, comparison to CYT treatment). Cyclosporine (CSA) was the positive control. (**D**–**R**) Immunohistochemistry staining (40× magnification). Tissues were stained for TSLP (red) and nuclei (blue) (see Methods).

The effects of IDL and IDC on lactate dehydrogenase (LDH) secretion and thymic stromal lymphopoietin protein (TSLP) production were evaluated using an ex vivo model in which skin explants were treated with IL-4, IL-13, TNF-α and IL-5 (Figure 6A). LDH secretion was not significantly impacted by cytokine treatment (*p* > 0.05; Figure 6B). Treatment of skin explants with cyclosporine (CSA) (positive control) decreased LDH secretion by 30% although this effect was not significant (*p* > 0.05; Figure 6B). Treatment with IDL led to a non-significant 17% decrease in LDH secretion, although a larger marginally significant 56% decrease in LDH secretion was seen with IDC treatment (*p* = 0.069, one-tailed two-sample *t*-test, Figure 6B).

The cytokine cocktail led to a significant 17% increase in TSLP production (*p* = 0.034, one-tailed two-sample *t*-test; Figure 6C–I). The elevated TSLP was decreased by 16% with CSA treatment, although this change was only marginally significant (*p* = 0.073, one-tailed two-sample *t*-test; Figure 6C,G–L). Treatment of cytokine-treated explants with IDL or IDC decreased TSLP production by 11% and 14%, respectively, although these effects were not statistically significant (*p* > 0.10, one-tailed two-sample *t*-tests; Figure 6C,M–R).

### 2.12. IDL and IDC Inhibit LDH Activity

**Figure 7:**
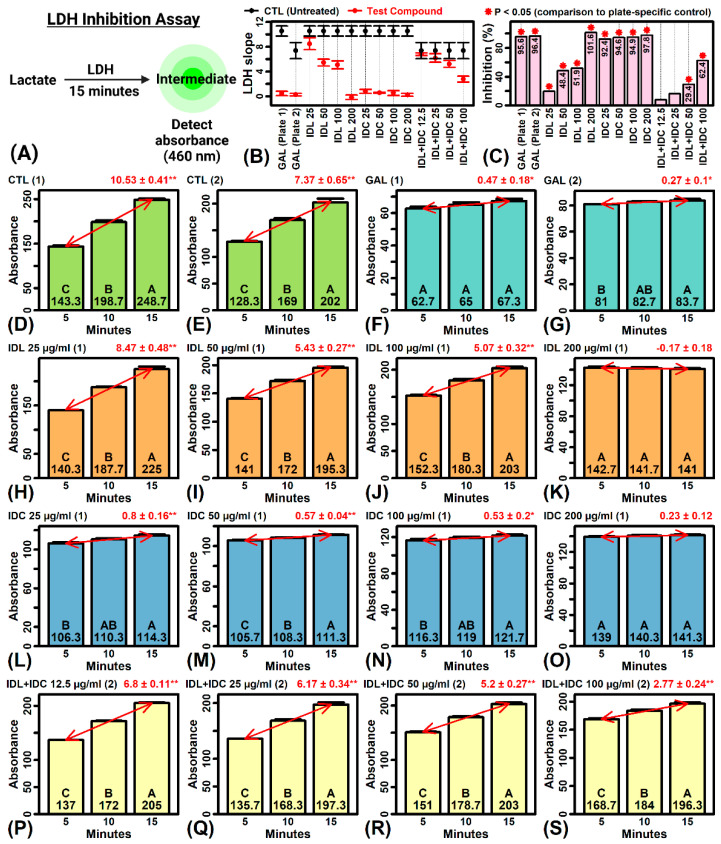
LDH inhibition assays. (**A**) Assay design. Lactate was converted to a fluorescent intermediate with absorbance detected at 460 nm. The reaction was allowed to proceed for 15 min, with increased absorbance (slope > 0) indicative of LDH activity. (**B**) Slope comparison (test compounds vs. CTL). Slope estimates ± 2 standard errors are plotted along with their plate-specific control. (**C**) Percent inhibition [(1-(treatment slope/CTL slope)) × 100]. The asterisk (*) is used to indicate a significant difference between treatment and CTL slope estimates (*p* < 0.05, linear model two-factor interaction effect). (**D**–**S**) All slope estimates. Bar graphs show average absorbance (±1 standard error) at each time point (*n* = 3 replicates). The least-square slope estimate is shown (upper right, ±1 standard error, * *p* < 0.05; ** *p* < 0.01). Bars that do not share the same letter differ significantly (*p* < 0.05, Fisher’s LSD). Assays were performed using one of two plates as indicated (parentheses, **upper-left**).

An in vitro assay was used to evaluate direct effects of IDL, IDC and IDL + IDC on LDH activity (Figure 7A). In the absence of any inhibitor, LDH catalyzed the conversion of lactate to a fluorescent intermediate, whose abundance could be detected based upon its absorbance at a wavelength of 460 nm (Figure 7D,E). This reaction was inhibited almost completely by galloflavin (GAL), which was used as the positive control inhibitor (Figure 7F,G) [35]. The addition of IDL to the reaction led to dose-dependent LDH inhibition (*p* < 0.05), with high-dose IDL (200 µg/mL) having an inhibitory effect equal to that of galloflavin (Figure 7B,C,H–K). On the other hand, addition of IDC led to almost complete LDH inhibition (90–100%) at all doses tested (25–200 µg/mL) (*p* < 0.05; Figure 7B,C,L–O). The combination IDL + IDC had a dose-dependent inhibitory effect on LDH activity (*p* < 0.05 for dose ≥ 50 µg/mL), with the total inhibitory effect similar to that observed for IDL but less than that seen for IDC (Figure 7C,P–S).

## 3. Discussion

**Figure 8:**
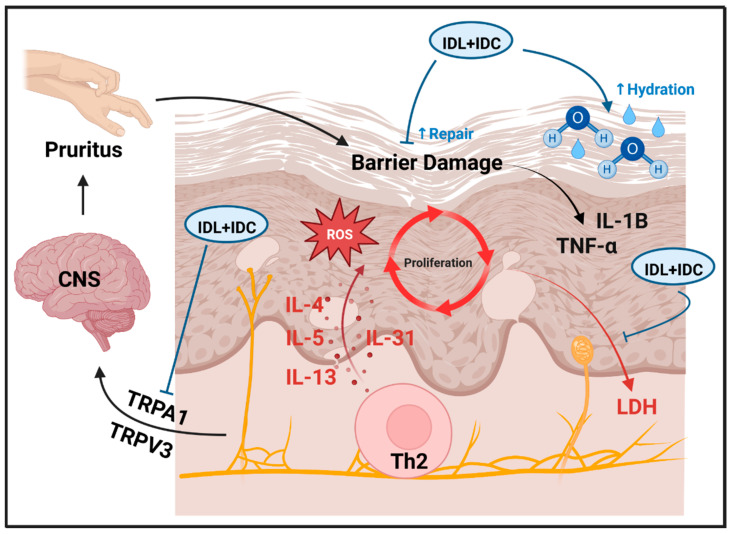
Hypothesized mechanisms of IDL + IDC in AD skin lesions. Th2 cells release IL-4, IL-5, IL-13 and IL-31 during the acute phase to initiate AD lesional development with skin barrier breakdown and transepidermal water loss. This promotes ROS accumulation and increased KC turnover with LDH leakage from the epidermis into the serum. Cutaneous nerves propagate signals mediated by TRPA1 to the CNS triggering pruritus and skin itching, leading to further skin barrier breakdown. IDL + IDC provide fatty acids directly to the *stratum corneum* to attenuate damage and limit barrier compromise, resulting in improved water retention and epidermal hydration. Improvement in barrier integrity limits local accumulation of pro-inflammatory cytokines such as TNF-α and IL-1B. IDL + IDC also down-regulates *TRPA1* and *TRPV3* expression, limiting neurogenic feedback to the CNS and further pruritus and excoriation. This reduces epidermal turnover within lesions resulting in reduced leakage of LDH from the cutaneous compartment into the systemic circulation.

Topical therapies are a mainstay treatment for atopic dermatitis (AD), particularly for children or those with mild-to-moderate disease who may not qualify for systemic immunosuppressive medications. Development of new topical compounds has therefore accelerated in order to broaden treatment options available for AD management [30]. Existing products, however, vary in their ability to protect and restore the epidermal barrier, and some over-the-counter moisturizers may even be harmful [36]. This study evaluated two isosorbide fatty acid diester compounds, isosorbide di-(linoleate/oleate) (IDL) and isosorbide dicaprylate (IDC), which have demonstrated clinical efficacy in human studies [12,13]. Both compounds were shown to bolster skin hydration and reinforce the epidermal barrier in human subjects, but underlying mechanisms have remained unclear and no prior study had evaluated effects of IDL + IDC used in combination. This study used validated AD laboratory models [15,16] to evaluate cellular and molecular responses to IDL, IDC and IDL + IDC. Our results suggest that effects of topically applied fatty acid diesters are not limited to barrier repair but may include inhibition of inflammatory cascades that amplify pruritus and cutaneous eruption (Figure 8).

Cytokines are key mediators of AD pathophysiology, with Th2 cytokine activity predominant during the acute phase (e.g., IL-4, IL-5, IL-13, IL-31) [37], and Th1, Th17 and Th22 developing a role during the chronic phase [38,39,40,41,42]. Previously, the combination of Th2 cytokines (IL-4 + IL + 13 + IL-31) with pro-inflammatory TNF-α was reported to induce an AD-like phenotype in tissue engineered human skin equivalents, characterized by spongiosis, altered KC differentiation, and changes in *stratum corneum* lipid composition [16]. We evaluated gene expression responses to this cytokine cocktail using a human skin equivalent model. The cocktail down-regulated expression of genes linked to epidermal development and cornification (Figure 2H,J), with decreased expression of genes encoding late differentiation proteins (i.e., *IVL*, *TGM1*; Figure S1A). Additionally, we observed increased expression of genes associated with specific cell cycle phases, including G1/S, S, G2 and M phases (Figure S3). These effects appear consistent with prior work, which demonstrated delayed epidermal differentiation, shifts in epidermal lipid composition, and increased basal cell proliferation in skin equivalents treated with Th2 cytokines and TNF-α [16]. Interestingly, however, the most robust response was up-regulated expression of genes localized to the mitochondrial inner membrane and respiratory chain complex, with many such genes functioning within the electron transport chain (Figure 2G,I). This effect had not been described previously but may mimic a feature of non-lesional AD skin, which has been characterized as having elevated mitochondrial activity with increased oxidative stress [43]. Our evaluation of the Th2/TNF-α AD skin model thus uncovered a new point of correspondence between model and disease phenotypes.

Genetic studies of AD have highlighted defects in barrier repair as a trigger leading to inflammatory cascades and downstream cytokine activation [44,45,46]. The use of occlusive moisturizers and other forms of barrier repair therapy has therefore been a backbone of AD treatment to limit the frequency and intensity of such inflammatory cascades [47]. In this study, IDL and IDC did not counter gene expression responses linked to Th2 cytokines (Figure 4). However, both compounds broadly reversed TNF-α-induced transcriptome changes and decreased expression of *IL1B* mRNA. The mRNA abundance of *IL1B* and *TNF* are each up-regulated following epidermal barrier damage [48,49,50,51], whereas occlusive treatments to restore barrier function normalize *IL1B* and *TNF* expression [51,52,53]. The role of these pathways in AD barrier physiology is not fully understood. Mice lacking IL-1 receptor exhibit accelerated barrier repair, suggesting that signaling through this pathway interferes with barrier recovery [54]. Although TNF-α was reported to increase SC ceramide levels [55], TNF-α also appears to induce AD-like changes in SC composition, leading to decreased abundance of cholesterol and long chain free fatty acids [16]. TNF-α was also reported to decrease abundance of loricrin and involucrin, which are key skin barrier proteins regulated by epidermal differentiation pathways [56]. Moreover, work done using cultured KCs showed that while IL-1B and TNF-α elicit short term improvements in barrier integrity, prolonged exposure increases membrane permeability [57]. These findings suggest mechanisms by which topical IDL and IDC may strengthen the epidermal barrier and enhance its recovery. By providing occlusive reinforcement, IDL and IDC may limit activation of the IL-1B and TNF-α pathways, preventing secondary damage stemming from activation of these cytokines (Figure 8).

IDL and IDC inhibited LDH activity in this study (IDC > IDL) and IDC treatment led to >50% reduction of LDH secretion by cytokine-stimulated skin explants. LDH is a cytosolic enzyme that catalyzes interconversion of lactate and pyruvate and is therefore essential to glycolysis and ATP production. In AD patients, serum LDH is associated with disease severity [58,59,60,61,62,63,64] and is predictive of treatment response [65,66,67,68]. LDH is viewed as a marker of tissue turnover and is present in epidermis and dermis with LDH5 being the dominant isoenzyme [69]. The activity of the LDH enzyme is itself increased in AD epidermis [64] and serum LDH levels have been associated with cutaneous inflammatory responses, such as increased abundance of kallikrein proteins in the AD *stratum corneum* [70]. The source of serum LDH in AD patients is therefore at least partly epidermal, although LDH may also be generated from immune cells [71]. The trend towards reduced LDH secretion by IDC-treated skin explants may indicate a protective effect, with less epidermal cell damage, along with slowing of epidermal turnover secondary to inflammation and mitogenic stimuli. On the other hand, our in vitro assays demonstrated direct LDH inhibition by IDL and IDC, which may confer an anti-proliferative effect, similar to that seen in malignant cells treated with LDH inhibitors [72]. Such effects may stabilize the AD epidermis to attenuate disease activity. This may be particularly important in the subset of patients with high serum LDH, who appear less likely to show long-term improvement with certain biologics such as dupilumab [65].

Transient receptor potential (TRP) channels act as cellular sensors expressed by nociceptive neurons [73] but are additionally expressed in immune cells [74] and skin cells such as KCs, melanocytes, and fibroblasts [75,76]. In this study, *TRPA1* expression was down-regulated by IDL + IDC in cytokine-treated RHE tissue (Figure 5F) and by IDL or IDL + IDC in cytokine-treated KCs (Figure 5L) (both results confirmed by RT-PCR). Additionally, expression of TRP vanilloid channel 3 (*TRPV3*) [77] was down-regulated about 40% by IDL and IDC, respectively, in RHE skin (Figure S6G). TRPA1 encodes a non-selective TRP cation channel that functions as an itch mediator by relaying signals to the central nervous system, by coordinating neurogenic inflammation [78], and by facilitating nonhistaminergic cutaneous dysregulation [79,80]. *TRPA1* has increased abundance in AD skin lesions [81] and its importance has been established from studies using multiple AD mouse models [81,82,83]. Treatment of mouse skin with 2,4-dinitrochlorobenzene (DNCB), for example, activates TRPA1 and generates an AD-like phenotype [82], and mice lacking TRPA1 have decreased dermatitis and pruritus scores, Th2 cytokines, epidermal hyperplasia, ear thickness, mast cell and macrophage infiltration [83]. Similarly, TRPA1 inhibition decreases itch-evoked scratching in IL-13-transgenic mice [81]. TRPA1 also appears to mediate itch triggered by diverse pruritogenic stimuli, such as periostin [84], bile acids [85], lysophosphatidic acid [86] and serotonin [87,88]. In skin, TRPA1 appears to facilitate neuro-immune interactions [89] and dysregulated calcium signaling seen in epidermal nerves following epidermal barrier impairment [90]. Its expression is seen in the basal epidermis, dermis, and hair follicle epithelium [75]. Interestingly, treatment of KCs with a TRPA1 agonist (icilin) increases expression of IL-1α and IL-1β mRNA and alters expression of genes associated with KC differentiation and proliferation [75]. Several other TRPA1 agonists were in fact reported to accelerate epidermal barrier recovery [91]. These studies suggest that down-regulation of *TRPA1* by IDL + IDC may inhibit nonhistaminergic pruritus, possibly by modifying signals generated from cutaneous nerves (Figure 8). Additionally, however, TRPA1 inhibition may have anti-inflammatory effects within the epidermis and influence barrier formation.

Our study has several limitations. First, this was an industry-sponsored study, which may increase risk of unrecognized bias in favor of the test products (IDL and IDC) [92]. Independent studies by third-party investigators may be useful in future work to confirm findings from this report. Second, laboratory investigators performing experiments in this study were not blinded with regard to the identity of test compounds, which may increase risk of unrecognized observer bias [93]. Third, this was a pre-clinical study that utilized laboratory-based model systems [15,16]. The current findings are hypothesis-generating and as such we have proposed candidate mechanisms of action (Figure 8). However, randomized placebo-controlled trials enrolling human subjects would be needed to demonstrate clinical efficacy.

The disease burden of AD is substantial and includes direct medical costs, personal costs, work productivity loses and impaired psychosocial functioning [94]. Aside from its cutaneous manifestations, AD has been associated with depression [95], anxiety [96], insomnia [97], obsessive compulsive disorder [98], decreased physical activity [99] and alcohol abuse [100]. This study has identified new mechanisms by which isosorbide fatty acid diesters may interrupt positive feedback cycles that drive xerosis, pruritus, scratching-induced eczema, excoriation and lichenification in AD skin. Our results therefore suggest ways in which “outside-in” topical therapies can complement “inside-out” systemic immunosuppressive medications as a multifaceted treatment approach to target cutaneous pathways that predispose to AD development.

## 4. Materials and Methods

### 4.1. Test Materials

IDL [Isosorbide di-(linoleate/oleate)] is commercially available from Sytheon (Parsippany, NJ, USA) under the trade name HydraSynol^®^ IDL (INCI: Isosorbide Disunflowerseedate; CAS no. 1818326-42-9). The composition of IDL consisted of approximately 70% linoleate and 15% oleate with other minor fatty acid esters. IDC [Isosorbide dicaprylate] is also commercially available from Sytheon under the trade name HydraSynol^®^ DOI (INCI: Isosorbide Dicaprylate; CAS no. 64896-70-4). The composition of IDC consisted of >99% mono- and di-caprylic acid esters of isosorbide with >95% diester content.

### 4.2. RHE Tissue Culture

Pre-mature RHE tissues (4 days post airlift) were obtained from a commercial provider (Zen-Bio, Durham, NC, USA) and maturation was continued in Zen-Bio Airlift Media (lot no. 011722) until day 11. On day 11, Zen-Bio Airlift Media was replaced with Zen-Bio assay media (lot. no. 042922) and test compounds (IDL and/or IDC) were applied topically at 10 mg/cm^2^. All compounds were prepared in caprylic/capric triglyceride (CCT) vehicle solution. Control (CTL) tissues were treated only with vehicle solution. Cultures were continued until day 15. On day 7 of air exposure, IL-4, IL-13, IL-31 and TNF-α were added to the culture medium at concentrations of 30 ng/mL, 30 ng/mL, 15 ng/mL and 3.5 ng/mL, respectively. Cytokines were obtained from R&D Systems (Minneapolis, MN, USA) and medium was changed every 2 days. TEER measurements were obtained on the final day of the experiment using the EVOM2 Epithelial Volt/Ohm Meter with STX2 electrode (World Precision Instruments, Sarasota, FL, USA). Following TEER measurements, tissues were divided into two equal parts, with one section fixed in formalin for histochemical analysis and the other section preserved in RNA-later.

### 4.3. Microarray Analyses

Microarray analyses were performed on RHE samples treated with caprylic/capric triglyceride vehicle only (CTL), cytokine cocktail (CYT), cytokines with 4% IDL (CYT + IDL), cytokines with 4% IDC (CYT + IDC) or cytokines with 2% IDL and 2% IDC (CYT + IDL + IDC) (*n* = 3 replicates per group). Microarray hybridizations were performed by Thermo Fisher Scientific (Santa Clara, CA, USA) using the Clariom S platform with standard protocols. The data analysis was performed using 15 raw CEL files. Inspection of microarray pseudoimages did not demonstrate evidence for prominent spatial artifacts (Figure S7A–O). RNA 260/280 absorbance ratios were approximately 2.0 for all samples and no greater than 2.12, consistent with high-purity RNA (Figure S7P). Eukaryotic hybridization spike controls were detected in each sample at appropriate levels reflecting their concentration gradient (Figure S7Q). Likewise, polyadenylated labeling controls were present in all samples with expected differences in expression (Figure S7R). Area under the curve (AUC) statistics were near 1.00 for all samples, consistent with good separation between signals arising from probes targeting intronic and exonic gene regions (Figure S7S). Normalized unscaled standard error (NUSE) median and interquartile range (IQR) values [101] were within an acceptable range, except for sample CTL-2 which had elevated NUSE median and IQR (Figure S7T,U). Likewise, relative log expression (RLE) median and IQR [101] were elevated for CTL-2 but otherwise acceptable for other samples (Figure S7V,W).

Normalization was performed using the robust multichip average (RMA) method (R package: oligo, function: rma) [102]. This yielded expression intensities for 27,189 probe sets. Probe sets lacking gene symbol annotation were excluding, yielding 21,448 annotated probe sets. Of these, we included only 19,937 probe sets associated with protein-coding genes. Among these, there were some “sibling” probe sets annotated with the same gene symbol [103]. In such cases, we included only one probe set for each symbol having the highest average expression across all samples. Following this filter, there remained 18,088 probe sets upon which further analyses were based, where each probe set was uniquely associated with a human protein-coding gene. Hierarchical cluster analysis showed that CTL samples grouped apart from all others, with smaller differences among cytokine-treated samples and no strong evidence for outliers (Figure S7X). Consistent with this, CTL samples differed from others with respect to principal component (PC) axis 1, whereas cytokine-treated samples co-localized in the bivariate PC space (Figure S7Y).

### 4.4. Differential Expression Analyses

Differential expression analyses were carried out with four comparisons (CYT vs. CTL, CYT + IDL vs. CYT, CYT + IDC vs. CYT and CYT + IDL + IDC vs. CYT). Of the 18,088 genes included in the analysis, differential expression testing was performed using only those genes with detectable expression in at least 2 of the 6 samples involved in a given comparison. A gene was considered to have detectable expression if its normalized signal intensity was above the 20th percentile among the 18,088 included protein-coding genes. Additionally, we excluded genes with low variation in gene expression. To identify such genes, the standard deviation of normalized expression intensity estimates was calculated among the 6 samples involved in a given comparison, and genes with standard deviation less than the 5th percentile were excluded. These filters removed about 20% of protein-coding genes from further analyses. Differential expression testing was thus performed upon 14,347, 14,156, 14,161 and 14,171 genes with respect to the CYT vs. CTL, CYT + IDL vs. CYT, CYT + IDC vs. CYT and CYT + IDL + IDC vs. CYT comparisons, respectively. Differential expression testing was performed using linear models with empirical Bayes moderated t-statistics [104] (R package: limma, R functions: lmFit and eBayes). To control the false discovery rate (FDR) among the 14,156 to 14,347 genes included in each analysis, raw *p*-values were adjusted using the Benjamini-Hochberg method [105].

A larger number of genes were associated with extreme moderated T statistics for the CYT vs. CTL comparison (Figure S8A–D), consistent with a stronger treatment effect and greater differential expression. However, *p*-value distributions were left-shifted for each comparison (Figure S8E–H) and *p*-value empirical CDFs differed significantly from linearity (*p* < 0.05, Kolmogorov–Smirnov test; Figure S8I–L). In each case the number of increased DEGs was larger than the number of decreased DEGs, although volcano plots were roughly symmetrical (Figure S8M–P). Differential expression FC estimates did not vary systematically between low- and high-expressed genes (Figure S8Q–T).

### 4.5. Cytokine-Stimulated Skin Explants

Full-thickness human skin (30 cm^2^, phototype 3) without stretch marks was obtained from surgical waste (abdominoplasty) of a female Caucasian donor who provided written informed consent. The sample was received on ice in sterile gauze and immediately processed with removal of adipose tissue and replicate 10 mm punch biopsies. Each punch biopsy was placed in a cell culture plate with the dermal part immersed in medium (cat. no. MIL215C, batch no. MIL215008, Biopredic International, Saint-Grégoire, France) and the epidermal surface in contact with air. A total of 15 punch biopsies were assigned to five conditions (3 replicates/condition). Conditions were no treatment (CTL), treatment with cytokine cocktail (CYT), treatment with cytokines and cyclosporine (CYT + CSA), treatment with cytokines and IDL (CYT + IDL), and treatment with cytokines and IDC (CYT + IDC). The cytokine cocktail (IL-4 + IL-13 + TNF-α + IL-5) was added at a concentration of 200 ng/mL. The immunosuppressant cyclosporine was used as a positive control and added to medium at a concentration of 1 μM. Test compounds (IDL and IDC) were diluted in culture medium at a concentration of 50 μg/mL. Following 24 h, culture medium was collected and biopsies were placed in Optimum Cutting Temperature (OCT) medium and cryopreserved at a temperature of −80 °C.

### 4.6. LDH Detection Assay

The LDH detection assay was performed using culture medium obtained on the final day of the experiment. A commercial LDH assay kit was purchased (Thermo Fisher Scientific, Waltham, MA, USA) and assays were performed following the manufacturer’s instructions. A negative control (blank) was done using fresh culture medium without tissue. Optical densities from the blank were used to normalize those obtained from other samples. A positive control test was performed using Triton 0.1%, which yielded LDH release with an optical density 2-fold higher than any other test sample.

### 4.7. TSLP Assays

Cryopreserved biopsies were sectioned to a thickness of 8 μm within a cryostat cabinet at −25 °C. Biopsies were mounted on polylysine superfrost slides (Thermo Fisher Superfrost Plus) and returned to a temperature of −80 °C for storage. Slides were thawed and fixed in formalin for immunostaining. To prevent non-specific primary antibody binding, slides were incubated in 0.2% Triton X-100 in PBS (5 min) followed by PBS 3% BSA and 0.1% tween (30 min). Slides were then incubated overnight (4 °C) with TSLP primary antibody (Abcam, cat. nos. ab47943 and ab188766). Slides were then incubated with anti-rabbit cyanine 5 (Jackson ImmunoResearch, West Grove, PA, USA) secondary antibody for 1 h at room temperature. In the final step, slides were incubated with Hoechst^®^ 33342 1/5000e (10 min) and preserved in mounting medium Fluoromount-G (Thermo Fisher Scientific, 00-4958-02). Images were obtained using an epifluorescence microscope with cyanine 5 channel (Zeiss, Axio Imager Z1, ApoTome, Zen2 blue edition software, Carl Zeiss Microscopy GmbH, Carl-Zeiss-Promenade 10, Jena, Germany). An ImageJ software (U.S. National Institutes of Health, Bethesda, MD, USA) macro was used for quantification of cytoplasmic TSLP. Only images having approximately 200 nuclei and intact epidermis were used (4–6 images per replicate). Processing steps included segmentation of the epidermis with exclusion of the *stratum corneum* (which can contain non-specific staining). The average cytoplasmic cyanine 5 intensity fluorescence was then calculated in the included region to estimate TSLP production.

### 4.8. LDH Inhibition Assay

The assay was performed using a commercial screening kit (Abcam, Cambridge, UK, cat no. 283393). Test materials were prepared in LDH assay buffer at twice their final desired concentration. Galloflavin was used as the positive control [35]. Samples were prepared in dimethyl sulfoxide (DMSO) with a final DMSO assay concentration of 5%. The untreated (CTL) group was DMSO alone. The assay was performed by combining 50 µL of test material with 40 µL of substrate/cofactor solution within wells of a 96-well plate. The reaction was started by adding 10 µL of LDH enzyme solution, and absorbance at 460 nm was then monitored at 5 min intervals using a plate reader. The reaction leads to accumulation of a substrate with absorbance at 460 nm when acted upon by LDH. The estimated rate of increased absorbance (slope) at 5, 10 and 15 min was thus used as a proxy for LDH activity, with percent inhibition calculated by comparing slope estimates between CTL and test sample assays.

## Data Availability

The whole genome microarray data have been submitted to Gene Expression Omnibus (GEO) and are available under the accession number GSE217468.

## Supplementary Materials

The following are available online at https://www.mdpi.com/article/10.3390/ijms232214307/s1.

## Abbreviations

AD: atopic dermatitis; AUC: area under the curve; BET: betamethasone; BP: biological process; CC: cell component; CCT: caprylic/capric triglyceride; CSA: cyclosporine; CTL: control; CYT: cytokine; DEG: differentially expressed gene; DMSO: dimethyl sulfoxide; DNCB: dinitrochlorobenzene; FDR: false discovery rate; FLG: filaggrin; GAL: galloflavin; GEO: gene expression omnibus; GO: gene ontology; IDC: isosorbide dicaprylate; IDL: isosorbide di-(linoleate/oleate); IQR: interquartile range; IVL: involucrin; KC: keratinocyte; LDH: lactate dehydrogenase; LSD: least significant difference; MADAD: meta-analysis derived atopic dermatitis; NHEK: normal human epidermal keratinocytes; NUSE: normalized unscaled standard error; OCT: optimum cutting temperature; PIM: pimecrolimus; RHE: reconstructed human epidermis; RLE: relative log expression; RMA: robust multichip average; RT-PCR: real time polymerase chain reaction; TEER: transepithelial electrical resistance; TEWL: transepidermal water loss; TRP: transient receptor potential; TSLP: thymic stromal lymphopoietin.
