# Supplementary material for: Isosorbide Fatty Acid Diesters Have Synergistic Anti-Inflammatory Effects in Cytokine-Induced Tissue Culture Models of Atopic Dermatitis
Source: Int J Mol Sci. 2022 Nov 18;23(22):14307. doi: 10.3390/ijms232214307 (PMC9696169; doi:10.3390/ijms232214307)
Supplement: Supplementary file 1 [file ijms-23-14307-s001.zip › ijms-1996445-supplementary.pdf]

Supplementary Material

Isosorbide fatty acid diesters have synergistic anti-inflammatory effects in cytokine-induced tissue culture models of atopic dermatitis

William R. Swindell[1§], Krzysztof Bojanowski[2], Ratan K. Chaudhuri[3]
[1]University of Texas Southwestern Medical Center, Department of Internal Medicine, Dallas, TX, 75390, USA.
[2]Sunny BioDiscovery Inc., Santa Paula, CA, USA.
[3]Sytheon, Parsippany, NJ, USA.
[§]Corresponding Author.

Correspondence:
Dr. William R. Swindell, DO, PhD
University of Texas Southwestern Medical Center
Department of Internal Medicine
6201 Harry Hines Blvd.
Dallas, Texas, 75390

Email Addresses:
WRS: William.Swindell@UTSouthwestern.edu (ORCiD: 0000-0001-8504-6363)
KB: kbojanowski@sunnybiodiscovery.com (ORCiD: 0000-0001-8692-050X)
RKC: ratan@sytheonltd.com (ORCiD: 0000-0001-5676-9933)

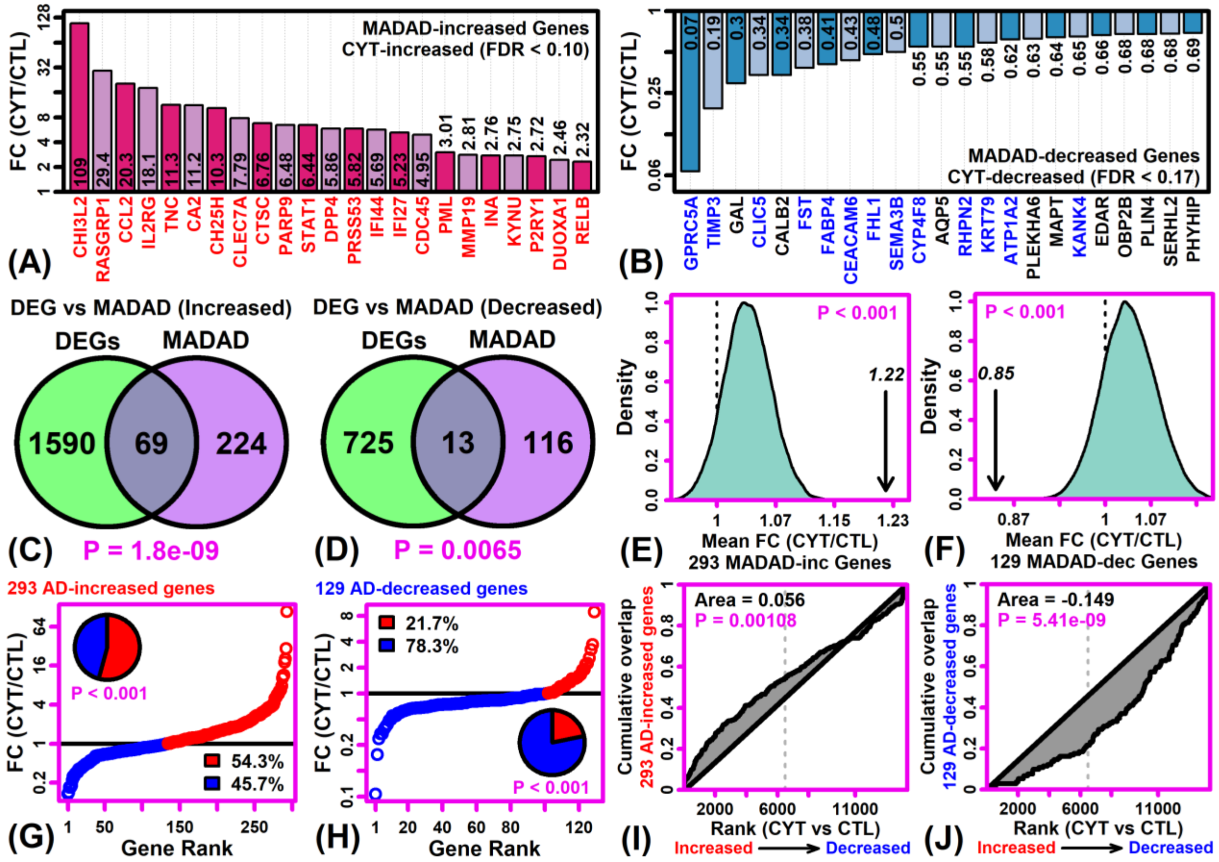

Figure S1. Comparison between cytokine-regulated genes and meta-analysis derived atopic dermatitis (MADAD) transcriptome. (A) CYT-increased/MADAD-increased genes. Genes significantly elevated by cytokine treatment (FDR < 0.10, FC > 1.50) are shown in red font. (B) CYT-decreased/MADAD-decreased genes. Genes significantly decreased by cytokine treatment (FDR < 0.10, FC < 0.67) are shown in blue font. (C) Overlap between CYT-increased DEGs (FDR < 0.10, FC > 1.50) and MADAD-increased genes. (D) Overlap between CYT-decreased DEGs (FDR < 0.10, FC < 0.67) and MADAD-decreased genes. In (C) and (D), the Fisher's exact test p-value assessing overlap significance is shown (bottom). (E) Average FC (CYT/CTL) among 293 MADAD-increased genes. (F) Average FC (CYT/CTL) among 129 MADAD-decreased genes. In (E) and (F), the average FC null distribution is shown (obtained by random sampling, n = 10000 simulations). The p-value is calculated by comparing the observed value (arrow) to null distribution outcomes. (G) Ordered FC estimates (CYT/CTL) among the 293 AD-increased genes. (H) Ordered FC estimates (CYT/CTL) among the 129 AD-decreased genes. In (G) and (H), the proportion of CYT-increased (red) and CYT-decreased (blue) genes is shown (pie chart) with p-value testing for increased vs. decreased imbalance (Fisher's exact test). (I, J) Gene set enrichment analyses. Part (I) shows cumulative overlap between the 293 MADAD-increased genes (vertical axis) and genes ranked based upon response to cytokine treatment (horizontal axis). Part (J) shows the cumulative overlap between 129 MADAD-decreased genes (vertical axis) and genes ranked based upon response to cytokine treatment (horizontal axis). The area between the cumulative overlap curve and diagonal is shown (area > 0: enrichment among cytokine-increased genes; area < 0: enrichment among cytokine-decreased genes; p-value: Wilcoxon rank sum test).

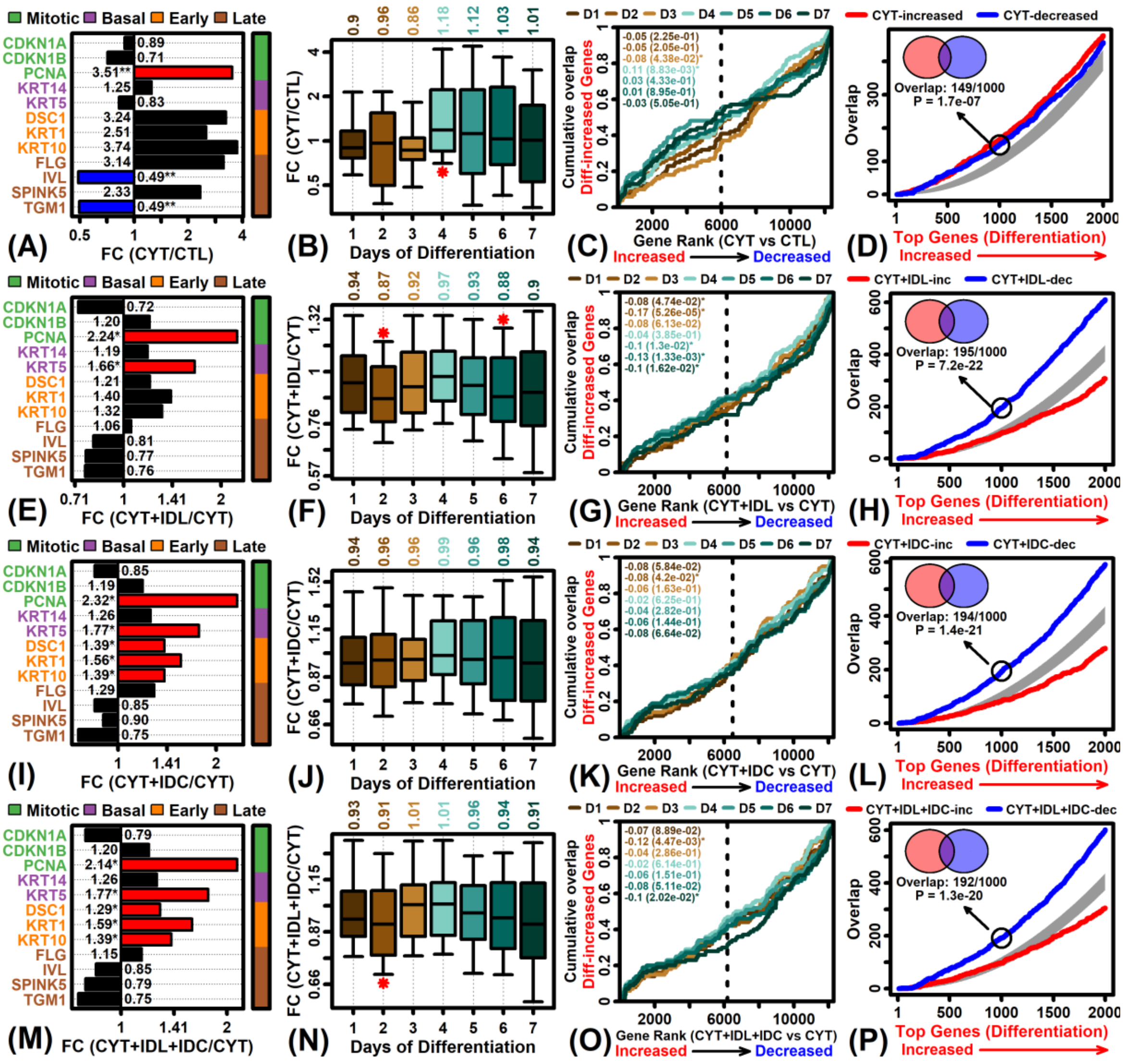

Figure S2. Genes associated with KC proliferation and differentiation. (A, E, I, M) Marker genes. FC estimates are shown for each gene (*P < 0.05, ** FDR < 0.10). (B, F, J, N) Average FC of the top 50 genes most strongly increased on each day of a regenerated epidermis time course (GSE52651). Boxplots outline the middle 50% of FC estimates (whiskers: 10th to 90th percentiles). An asterisk (*) is shown where the average FC estimate differs significantly from one (P < 0.05, Wilcoxon rank sum test). (C, G, K, O) GSEA analyses. Overlap is shown between the top 50 genes most strongly increased on each day of differentiation (vertical axis) and lists of genes ranked according to differential expression results (horizontal axis). AUC statistics are shown (top-left) with p-values in parentheses (AUC > 0: differentiation-increased genes enriched among genes increased in each differential expression analysis; AUC < 0: differentiation-increased genes enriched among genes decreased in each differential expression analysis). (D, H, L, P) Overlap between ranked gene lists. Overlap is shown between the top *n* differentiation-increased genes (1 < *n* < 2000) and the top *n* genes increased (red) or decreased (blue) in each differential expression analysis. Venn diagrams show overlap for the top 1000 genes (differentiation-increased vs. top 1000 genes decreased in each analysis; p-value: Fisher's exact test). Dark grey regions outline the 95% null distribution for the number of overlapping genes.

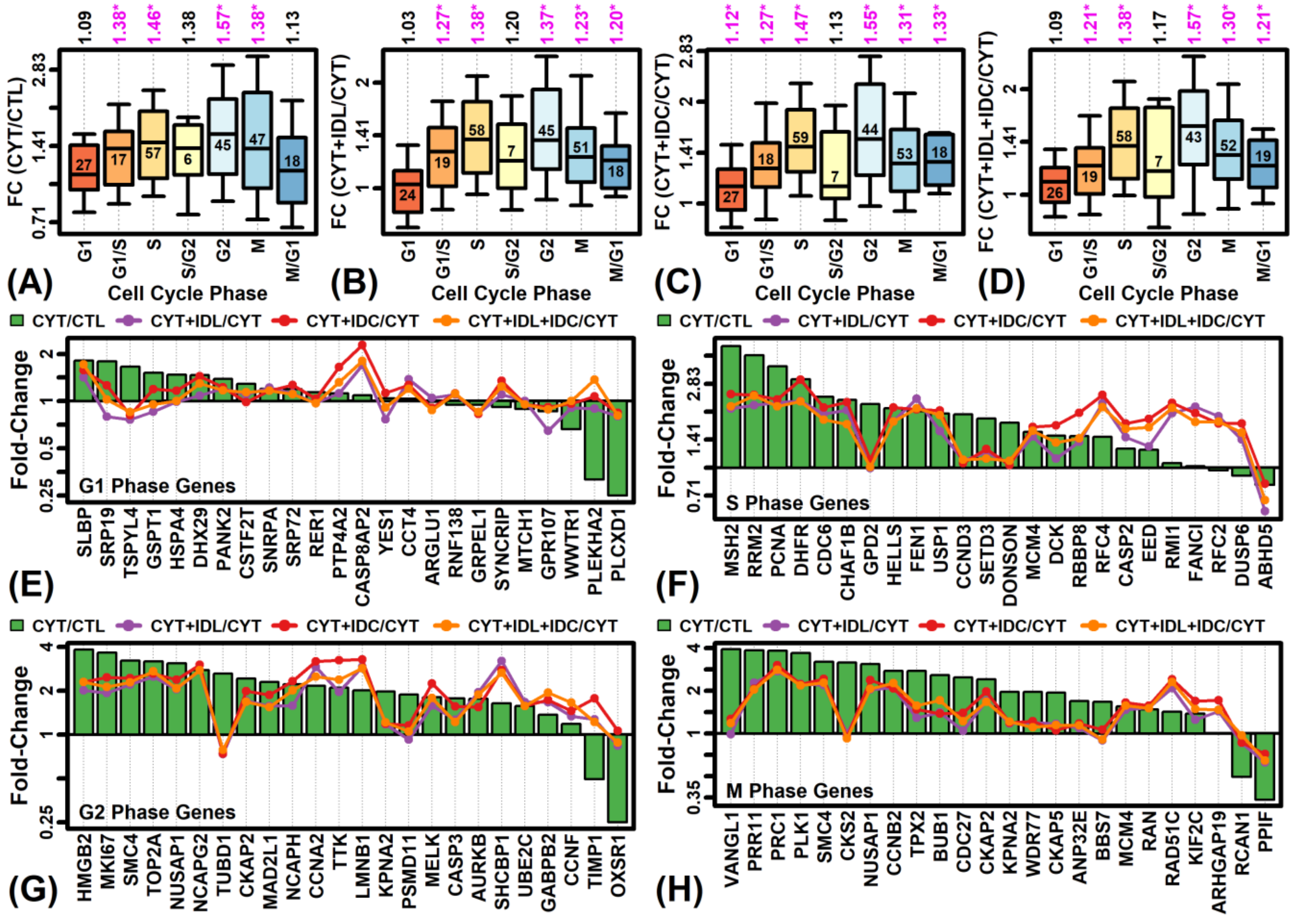

Figure S3. Cell cycle genes. (A - D) Average expression of genes associated with each cell cycle stage. Boxes outline the middle 50% of FC estimates for each gene set (whiskers: 10th to 90th percentiles). Median FC is shown in the upper margin (*P < 0.05, Wilcoxon rank sum test) and the number of genes analyzed is listed within each box. (E - H) G1, S, G2 and M phase marker genes. 24 marker genes are shown in each panel. These were selected by choosing those for which the value of min(p_1, p_2, p_3, p_4) is lowest, where p_1, p_2, p_3 and p_4 are p-values obtained from each of the 4 differential expression comparisons, respectively. Genes associated with each cell cycle phase were identified from the study of Mizuno et al. 2009 (BMC Genomics 10:137) [27].

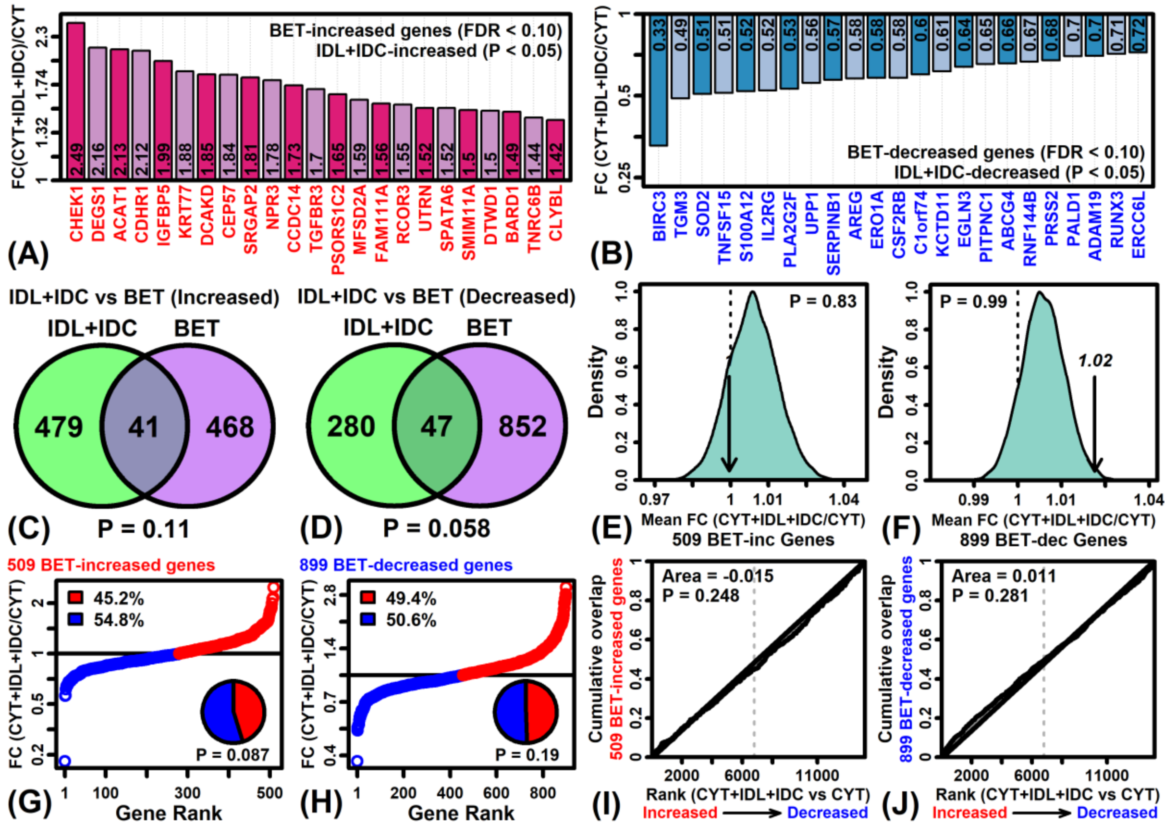

Figure S4. Comparison between IDL+IDC-regulated genes and genes altered in betamethasone (BET)-treated AD skin (GSE32473). (A) IDL+IDC-increased/BET-increased genes. Genes significantly elevated by IDL+IDC treatment (P < 0.05, FC > 1.50) are shown in red font. (B) IDL+IDC-decreased/BET-decreased genes. Genes significantly decreased by IDL+IDC treatment (P < 0.05, FC < 0.67) are shown in blue font. (C) Overlap between IDL+IDC-increased DEGs (P < 0.05, FC > 1.50) and BET-increased genes (FDR < 0.10, FC > 1.50). (D) Overlap between IDL+IDC-decreased DEGs (P < 0.05, FC < 0.67) and BET-decreased genes (FDR < 0.10, FC < 0.67). In (C) and (D), the Fisher's exact test p-value assessing overlap significance is shown (bottom). (E) Average FC (IDL+IDC+CYT/CYT) among 509 BET-increased genes. (F) Average FC (IDL+IDC+CYT/CYT) among 899 BET-decreased genes. In (E) and (F), the average FC null distribution is shown (obtained by random sampling, n = 10000 simulations). The p-value is calculated by comparing the observed value (arrow) to null distribution outcomes. (G) Ordered FC estimates (IDL+IDC+CYT/CYT) among the 509 BET-increased genes. (H) Ordered FC estimates (IDL+IDC+CYT/CYT) among the 899 BET-decreased genes. In (G) and (H), the proportion of IDL+IDC-increased (red) and IDL+IDC-decreased (blue) genes is shown (pie chart) with p-value testing for increased vs. decreased imbalance (Fisher's exact test). (I, J) Gene set enrichment analyses. Part (I) shows cumulative overlap between the 509 BET-increased genes (vertical axis) and genes ranked based upon response to IDL+IDC treatment (horizontal axis). Part (J) shows the cumulative overlap between 899 BET-decreased genes (vertical axis) and genes ranked based upon response to IDL+IDC treatment (horizontal axis). The area between the cumulative overlap curve and diagonal is shown (area > 0: enrichment among IDL+IDC-increased genes; area < 0: enrichment among IDL+IDC-decreased genes; p-value: Wilcoxon rank sum test).

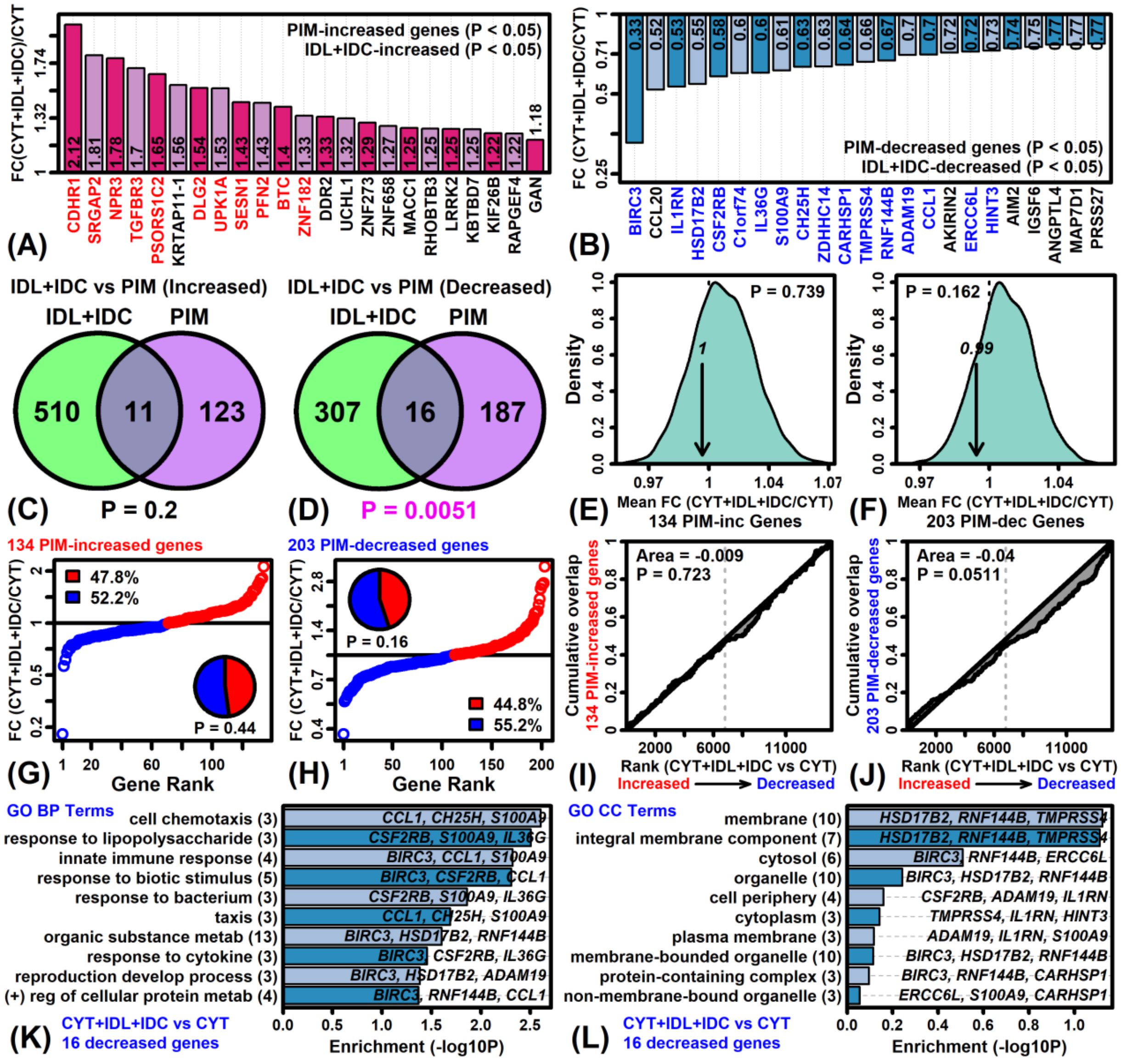

Figure S5. Comparison between IDL+IDC-regulated genes and genes altered in pimecrolimus (PIM)-treated AD skin (GSE32473). (A) IDL+IDC-increased/PIM-increased genes. Genes significantly elevated by IDL+IDC treatment ($P < 0.05$, $FC > 1.50$) are shown in red font. (B) IDL+IDC-decreased/PIM-decreased genes. Genes significantly decreased by IDL+IDC treatment ($P < 0.05$, $FC < 0.67$) are shown in blue font. (C) Overlap between IDL+IDC-increased DEGs ($P < 0.05$, $FC > 1.50$) and PIM-increased genes ($P < 0.05$, $FC > 1.50$). (D) Overlap between IDL+IDC-decreased DEGs ($P < 0.05$, $FC < 0.67$) and PIM-decreased genes ($P < 0.05$, $FC < 0.67$). In (C) and (D), the Fisher's exact test p-value assessing overlap significance is shown (bottom). (E) Average FC (IDL+IDC+CYT/CYT) among 134 PIM-increased genes. (F) Average FC (IDL+IDC+CYT/CYT) among 203 PIM-decreased genes. In (E) and (F), the average FC null distribution is shown (obtained by random sampling, n = 10000 simulations). The p-value is calculated by comparing the observed value (arrow) to null distribution outcomes. (G) Ordered FC estimates (IDL+IDC+CYT/CYT) among the 134 PIM-increased genes. (H) Ordered FC estimates (IDL+IDC+CYT/CYT) among the 203 PIM-

decreased genes. In (G) and (H), the proportion of IDL+IDC-increased (red) and IDL+IDC-decreased (blue) genes is shown (pie chart) with p-value testing for increased vs. decreased imbalance (Fisher's exact test). (I, J) Gene set enrichment analyses. Part (I) shows cumulative overlap between the 134 PIM-increased genes (vertical axis) and genes ranked based upon response to IDL+IDC treatment (horizontal axis). Part (J) shows the cumulative overlap between 203 PIM-decreased genes (vertical axis) and genes ranked based upon response to IDL+IDC treatment (horizontal axis). The area between the cumulative overlap curve and diagonal is shown (area > 0: enrichment among IDL+IDC-increased genes; area < 0: enrichment among IDL+IDC-decreased genes; p-value: Wilcoxon rank sum test). (K) GO BP terms enriched among the 16 IDL+IDC/PIM-increased genes. (L) GO CC terms enriched among the 16 IDL+IDC/PIM-decreased genes. In (K) and (L), the number of genes associated with each GO term is listed in parentheses and example genes are shown within each figure.

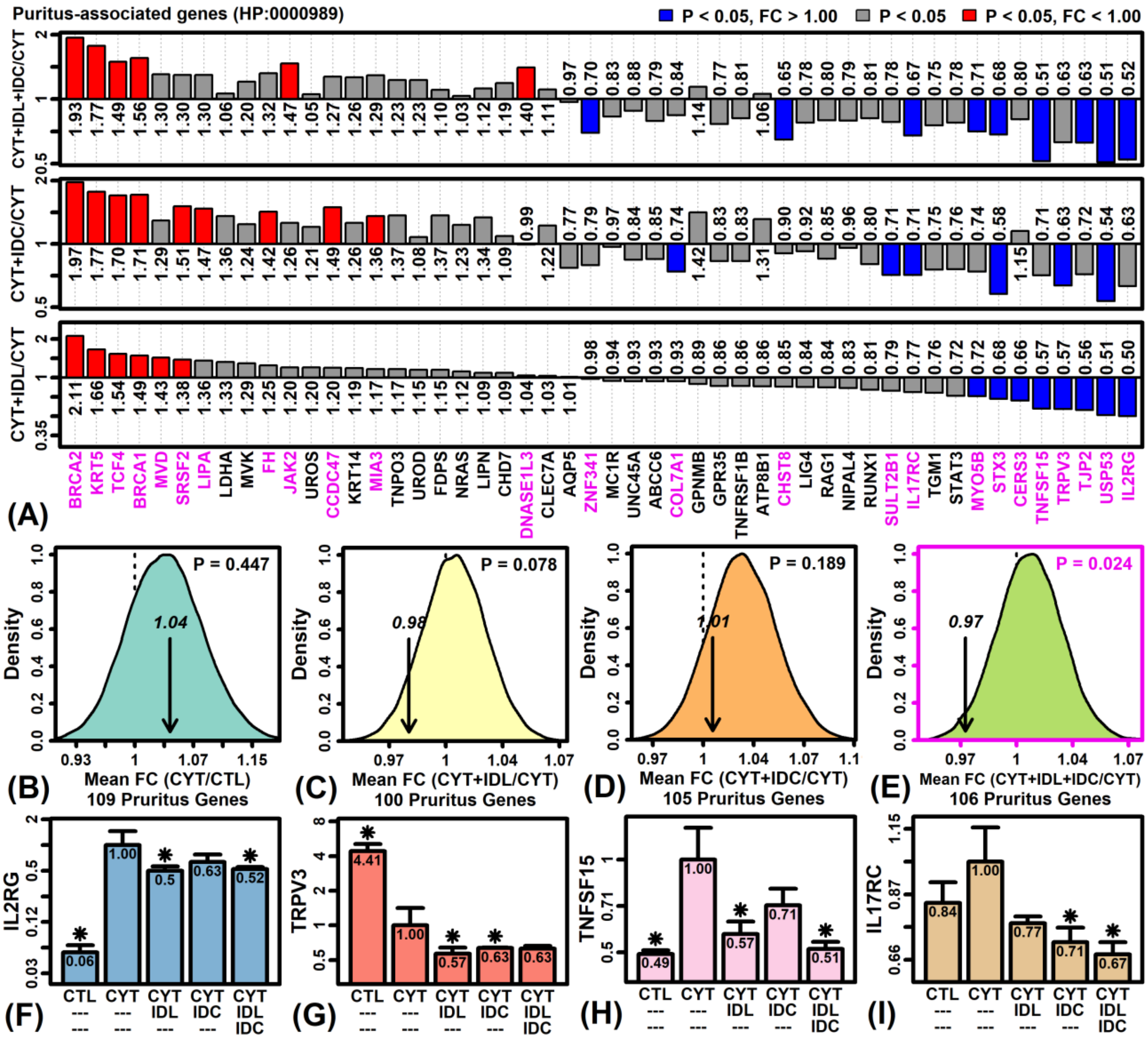

Figure S6. Pruritus-associated genes (Human Phenotype Ontology term HP:0000989). (A) Pruritus associated genes most strongly altered by IDL, IDC or IDL+IDC (top 50 genes shown). The FC is plotted for each gene and treatment. Gene labels with magenta font (bottom margin) were significantly altered by at least one of the three treatments. (B - E) Average FC among pruritus-associated genes. The average FC null distribution is shown (obtained by random sampling, *n* = 10000 simulations). The p-value is calculated by comparing the observed value (arrow) to null distribution outcomes. The number of pruritus-associated genes varies slightly in (B) - (E) since only genes with detectable expression in at least 2 samples were included in each differential expression analysis. (F) *IL2RG*. (G) *TRPV3*. (H) *TNFSF15*. (I) *IL17RC*. In (F) - (I), average expression is shown for each treatment (*P < 0.05, moderated t-test, comparison to CYT).

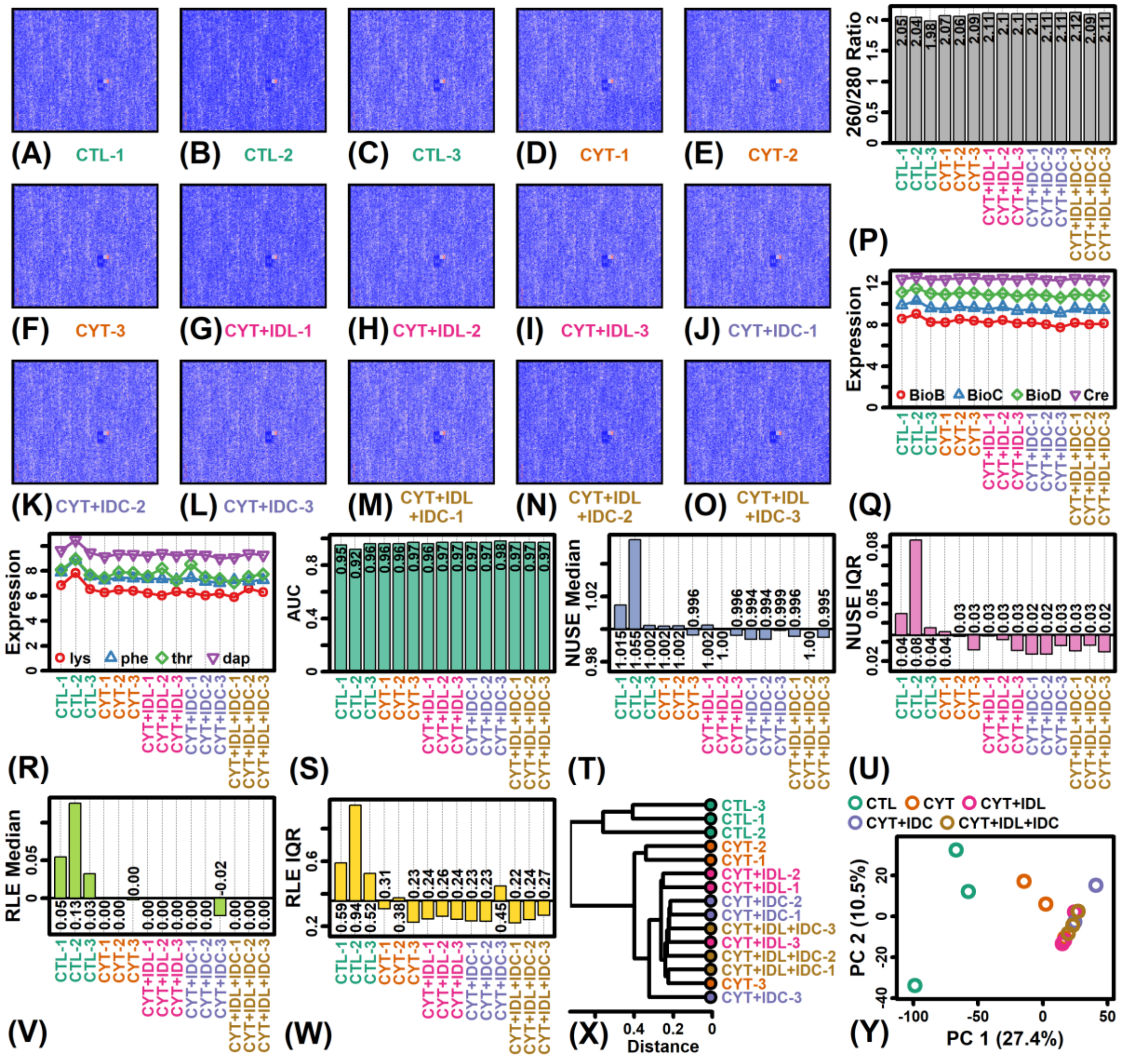

Figure S7. Quality control. (A - O) Microarray pseudoimages. Darker colors correspond to increased signal intensity (P) 260/280 absorbance ratios. (Q) Spike controls (Eukaryotic hybridization controls). Controls were spiked into the hybridization cocktail and used to evaluate hybridization efficiency. Concentrations vary among the four spike controls (BioB < BioC < BioD < Cre). (R) Polyadenylated in vitro synthesized RNA labeling controls (*B. subtilis* genes). Controls were added to RNA samples in staggered concentrations (lys < phe < thr < dap). (S) Area under the curve (AUC) statistics. Values are generated from the receiver operator curve (ROC) describing separation between intronic (true positives) and exonic (false positives) probes. Values near 1.0 correspond to expected separation of signals between probes targeting intronic and exonic gene regions. (T, U) Normalized unscaled standard error (NUSE) median and interquartile range (IQR). Larger NUSE median or NUSE IQR values correspond to lower quality arrays. (V, W) Relative log expression (RLE) median and IQR. RLE values deviating from zero or higher RLE IQR values may reflect lower quality arrays. (X) Cluster analysis. Samples were hierarchically clustered based upon expression across all samples (average linkage). (Y) Principal component plot. Samples are plotted with respect to the first two principal component axes.

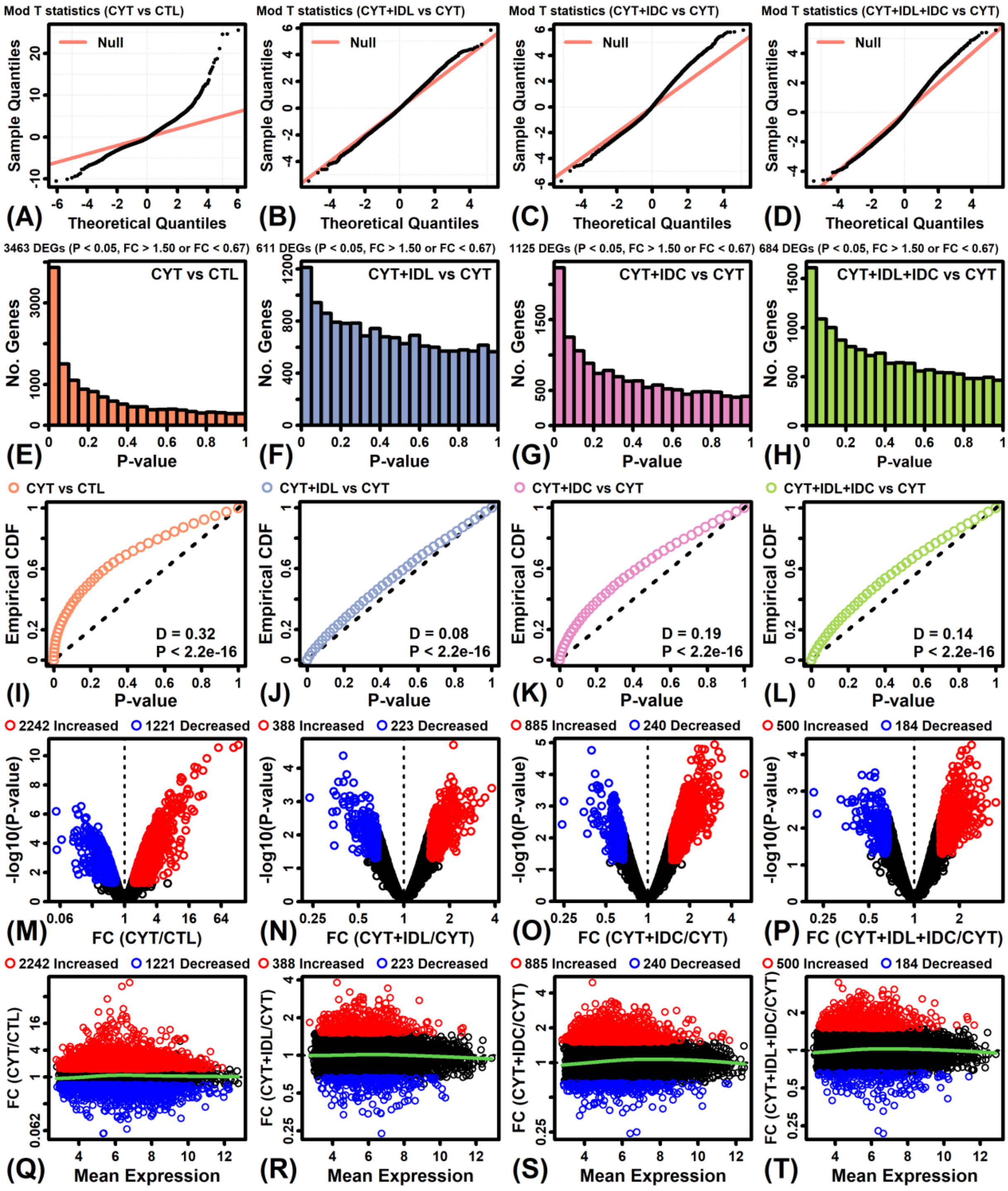

Figure S8. Differential expression analyses. (A - D) Quantile-Quantile plots. Moderated t-statistics are plotted against theoretical quantiles of the t-distribution. Deviation from a straight line is suggestive of differential expression. (E - H) Raw p-value distributions. The total number of DEGs (increased + decreased) identified for each comparison is shown (top margin). (I - L) Raw p-value empirical cumulative distribution functions (CDFs). The Kolmogorov-Smirnov test for non-linearity was performed and the resulting test statistic (D) and p-value are shown (bottom

right). (M - P) Volcano plots. -Log10-transformed p-values are plotted against FC estimates for each comparison. (Q - T) MA plots. FC estimates are plotted against average expression. The local polynomial regression (loess) fit is shown (green line). In (M) - (T), the number of increased (red) and decreased (blue) DEGs is shown (top margin, $P < 0.05$, $FC > 1.50$ or $FC < 0.67$).
